# A three-in-one-bullet for oesophageal cancer: replication fork collapse, spindle attachment failure and enhanced radiosensitivity generated by a ruthenium(ii) metallo-intercalator†
†Electronic supplementary information (ESI) available: Experimental methods, supplementary figures and tables. See DOI: 10.1039/c7sc03712k


**DOI:** 10.1039/c7sc03712k

**Published:** 2017-11-16

**Authors:** Martin R. Gill, Paul J. Jarman, Swagata Halder, Michael G. Walker, Hiwa K. Saeed, Jim A. Thomas, Carl Smythe, Kristijan Ramadan, Katherine A. Vallis

**Affiliations:** a CRUK/MRC Oxford Institute for Radiation Oncology , Department of Oncology , University of Oxford , Oxford , UK . Email: martin.gill@oncology.ox.ac.uk ; Email: katherine.vallis@oncology.ox.ac.uk; b Department of Chemistry , University of Sheffield , Sheffield , UK; c Department of Biomedical Science , University of Sheffield , Sheffield , UK

## Abstract

Substitutionally inert ruthenium(ii) polypyridyl complexes have been developed as DNA intercalating agents yet cellular DNA damage responses to this binding modality are largely unexplored. Here, we show the nuclear-targeting complex [Ru(phen)_2_(tpphz)]^2+^ (phen = 1,10-phenanthroline, tpphz = tetrapyridophenazine) generates rapid and pronounced stalling of replication fork progression in p53-deficient human oesophageal cancer cells. In response, replication stress and double-strand break (DSB) DNA damage response (DDR) pathways are activated and cell proliferation is inhibited by growth arrest. Moreover, mitotic progression is compromised by [Ru(phen)_2_(tpphz)]^2+^, where the generation of metaphase chromosome spindle attachment failure results in spindle assembly checkpoint (SAC) activation. This dual mechanism of action results in preferential growth inhibition of rapidly-proliferating oesophageal cancer cells with elevated mitotic indices. In addition to these single-agent effects, [Ru(phen)_2_(tpphz)]^2+^ functions as a radiosensitizer with efficiency comparable to cisplatin, which occurs through a synergistic enhancement of DNA damage. These results establish that DNA replication is the target for [Ru(phen)_2_(tpphz)]^2+^ and provide the first experimental evidence that ruthenium-based intercalation targets multiple genome integrity pathways in cancer cells, thereby achieving enhanced selectivity compared to existing DNA-damaging agents such as cisplatin.

## Abstract 2

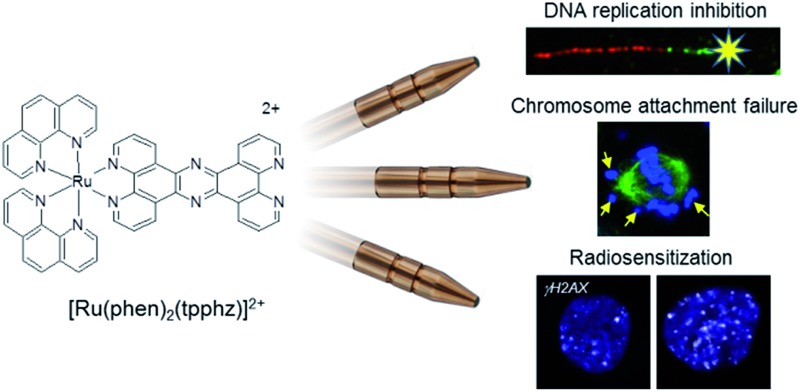
[Ru(phen)_2_(tpphz)]^2+^ simultaneously inhibits DNA replication, blocks mitosis and enhances DNA-damaging ionising radiation in oesophageal cancer cells.

## Introduction

Small molecules that interfere with DNA replication are widely-used anti-cancer drugs and are often employed in combination therapy alongside ionising radiation (IR) to treat cancer.^1^,^2^ One example of a clinical radiosensitizer is the platinum drug cisplatin, which generates both inter and intra-strand Pt–DNA cross-links and double-strand breaks (DSBs) that slow cell-cycle progression through S-phase, exacerbating IR-induced DNA damage.^3^,^4^ Although cisplatin-based chemoradiotherapy is highly effective in many cases, oesophageal cancers are marked by poor 5-year survival rates, typically <20%.^5^ Cisplatin is associated with nephrotoxicity which limits dose escalation and attempts to improve the outcome of patients with oesophageal cancer using alternative DNA-damaging chemotherapy such as doxorubicin have been unsuccessful.^6^ Application of these potent cytotoxic agents has also been hampered by the fact that the majority (77%) of oesophageal cancers lack p53 function^7^ and therefore possess a reduced capacity to activate apoptotic pathways in response to significant DNA damage.^8^ Thus, less toxic compounds that operate by alternative mechanisms of action and can also function as radiosensitizers are required.

As typified by [Ru(bpy)_2_(dppz)]^2+^ (bpy = 2,2-bipyridine, dppz = dipyridophenazine),^9^ substitutionally inert ruthenium(ii) polypyridyl complexes (RPCs) that interact with DNA solely *via* intercalation have been developed as site- and structure-specific luminescent DNA binding agents.^9^,^10^ Recent X-ray crystal structures have provided molecular insight into RPC metallo-intercalation in unprecedented detail.^11^–^16^ This includes evidence that [Ru(phen)_2_(dppz)]^2+^ (phen = 1,10-phenanthroline) adopts multiple intercalative geometries^12^ and Ru(dppz) semi-intercalation induces marked kinking of duplex DNA;^11^ a structural distortion similar to that observed following platination.^17^ Based on these studies with isolated DNA, the cellular uptake and anti-cancer properties of RPCs have become of increasing interest.^18^–^20^ However, despite the large number of DNA-interactive RPCs that have now been screened for anti-cancer activity,^21^ few have unequivocally been shown to have genomic DNA as their pharmacological target.^22^–^24^ As a result, cellular DNA damage responses to lesions generated by RPC mono-intercalation are completely unknown; a considerable barrier to therapeutic development of this class of DNA-binding agent. Moreover, while RPCs have generated much interest as photosensitizers for photodynamic therapy (PDT),^25^–^27^ studies of RPCs in combination with IR have been rare.^23^,^28^,^29^ This is surprising as radiotherapy is a mainstay of cancer medicine: high-energy X-rays or targeted radionuclide therapeutics have a far greater depth of penetration in tissue than visible light and radiotherapy is therefore capable of targeting a wider range of cancers.^2^,^30^

**Fig. 1:**
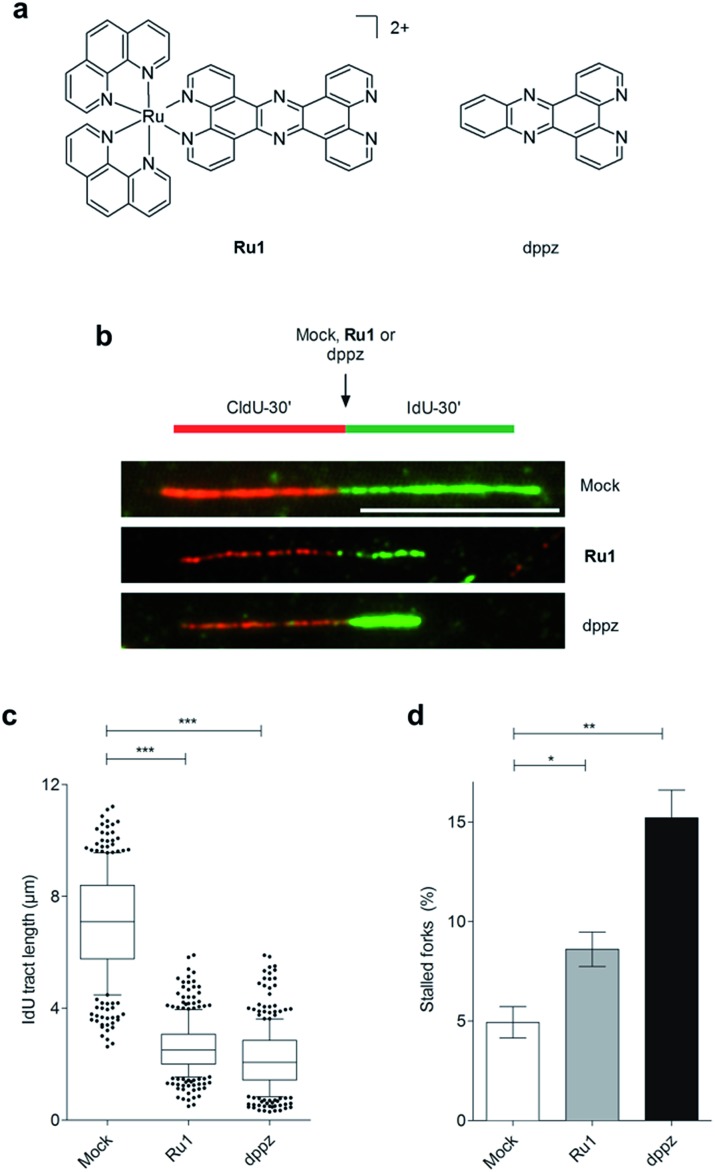
(a) Structures of **Ru1** and dppz. **Ru1** was used as the chloride salt and as a mixture of *Λ* and *Δ* enantiomers. (b) Representative images of DNA replication fibres in OE21 oesophageal cancer cells. CldU (first pulse, 30 min) incorporation is shown in red, incorporated IdU (second pulse, 30 min) in green. The second nucleotide (IdU) was either mock-treated or incubated in the presence of **Ru1** or dppz to determine impact on DNA replication fork progression. Scale bar = 10 μm. (c) Quantification of IdU-labelled tract length in the presence of **Ru1** (21 μM) or dppz (7 μM). Tract length was determined for >100 forks per condition. Whisker box plots show mean values and data within the 10–90 percentile. (d) Quantification of completely stalled forks (*i.e.* CldU tract only) for **Ru1** or dppz treatment as in (b). Fork stalling was quantified for >300 forks per condition and experiment. The experiment was repeated three times. Data were analysed using unpaired two-tailed *t* test (*P < 0.1, **P < 0.01, ***P < 0.005).

[Ru(phen)_2_(tpphz)]^2+^ (tpphz = tetrapyrido[3,2-*a*:2′,3′-*c*:3′′,2′′-*h*:2′′′,3′′′-*j*]phenazine), **Ru1** (Fig. 1a), is a water-soluble hydrophilic mono-intercalator (log *P* = –1.24, DNA *K*_b_ = 3 × 10^5^ M^–1^) that shows *in vitro* anti-cancer activity^31^ and toxicity has been established in murine models.^32^ Here, we present a detailed characterisation of the cellular response to DNA damage generated by **Ru1** in p53-deficient oesophageal cancer cells and explore the complex in combination with IR.

## Results and discussion

### Nuclear localisation of **Ru1**

Previous confocal laser scanning microscopy (CLSM) and transmission electron microscopy (TEM) studies indicated that **Ru1** targets nuclear DNA.^31^ ICP-MS (inductively coupled plasma mass spectrometry) analysis confirmed high (>70%) cellular ruthenium content in isolated nuclear fractions of **Ru1**-treated oesophageal cancer cells (Fig. S1 in the ESI†) while visualisation of intracellular MLCT (metal to ligand charge-transfer) emission of **Ru1** in OE21 oesophageal squamous cell carcinoma (ESCC) cells showed strong overlap with the DNA dye DAPI (Fig. S2†). In comparison, OE21 cells treated with [Ru(phen)_2_(dppz)]^2+^ (log *P* = –1.48 (ref. 18)) possessed a 13-fold lower average cellular Ru content and a 28-fold reduction in nuclear Ru content than cells treated with **Ru1** (Fig. S1a†). In addition to this, minimal nuclear MLCT emission in [Ru(phen)_2_(dppz)]^2+^-treated cells was evident by CLSM (Fig. S2†). These results indicate substantially greater cellular uptake and enhanced nuclear targeting are demonstrated by **Ru1** over [Ru(phen)_2_(dppz)]^2+^.

### **Ru1** stalls replication fork progression

On the basis of our recent discovery that the multi-intercalator [Ru(dppz)_2_(PIP)]^2+^ (PIP = 2-(phenyl)imidazo[4,5-*f*][1,10]phenanthroline) is able to stall replication forks,^23^ the ability of **Ru1** to similarly affect DNA replication was examined by DNA fibre assay. By sequential incorporation of halogenated nucleotides and immunofluorescence staining, this technique allows visualisation of the progression of individual replicating DNA strands.^33^,^34^ Accordingly, OE21 cells were pulse-labelled with the thymidine analogue CIdU for 30 min before treatment with **Ru1** and concomitant labelling with a second thymidine analogue (IdU) for an additional 30 min, thereby allowing examination of the direct impact of **Ru1** upon replication fork progression (Fig. 1b). Strikingly, the addition of 21 μM **Ru1** (the 24 h IC_50_ concentration, Table 1) in this manner resulted in a large decrease in median IdU tract length in DNA fibres, indicating extensive replication fork stalling generated due to the inclusion of the complex (Fig. 1c). This was accompanied by a 1.7-fold increase in completely stalled/terminated replication forks (CldU tract only – Fig. 1d). This marked impact on replication fork progression indicates pronounced and rapid DNA replication inhibition is generated directly by the addition of **Ru1**; an unprecedented result for a substitutionally inert metal complex that binds DNA solely by mono-intercalation.

**Table 1:** IC_50_ values (μM) of **Ru1**, dppz, cisplatin or [Ru(phen)_2_(dppz)]^2+^ towards OE21 human oesophageal squamous cell carcinoma, OE33 and FLO-1 human oesophageal adenocarcinoma cancer and hSAEC1-KT normal human small airway epithelial cells^*a*^

	OE21		OE33		FLO1		hSAEC1-KT	
	24 h	72 h	24 h	72 h	24 h	72 h	24 h	72 h
**Ru1**	21 ± 4.2	2.9 ± 1.5	44.5 ± 3.5	27 ± 2.8	42 ± 8	11.5 ± 2.1	78.5 ± 1.3	22 ± 2
dppz	6.5 ± 2.1	3.3 ± 0.4	6.6 ± 2	5.7 ± 0.9	7.5 ± 0.8	1.9 ± 1.1	>10	>10
Cisplatin	22.6 ± 3.6	6.3 ± 0.6	11 ± 1.4	3.25 ± 1	26.5 ± 0.7	4.7 ± 0.7	21.5 ± 5	15.8 ± 2
[Ru(phen)_2_(dppz)]^2+^	>100	>100	>100	>100	>100	34.0 ± 5.7	N.D.	N.D.

^*a*^Determined by MTT assay (24 or 72 h constant exposure). Data mean of two independent experiments ± S.D. N.D. = not done.

### DNA damage response (DDR) activation by **Ru1**

**Fig. 2:**
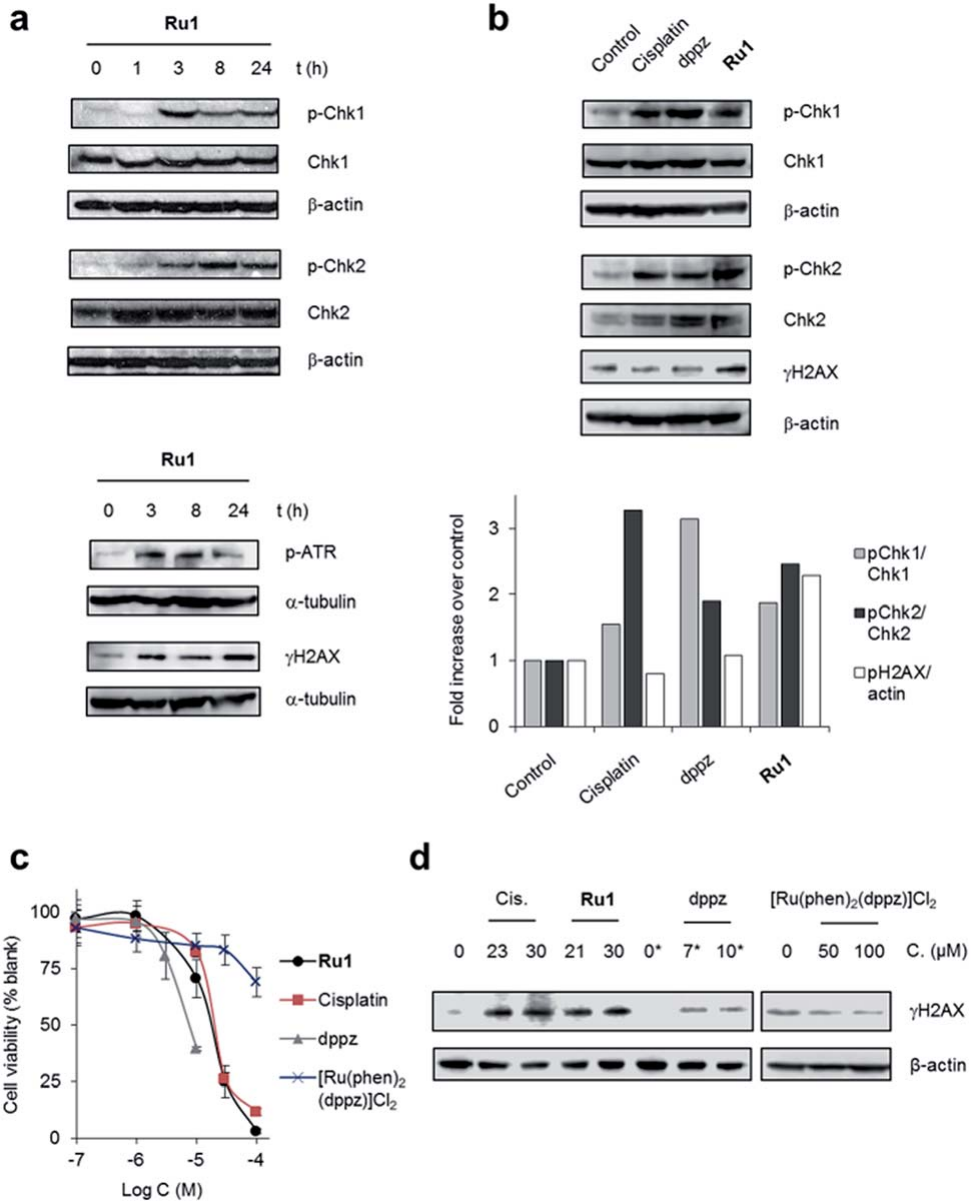
(a) DNA damage response (DDR) activation by **Ru1**. Whole-cell extracts of OE21 cells treated with **Ru1** (20 μM) for 1, 3, 8 or 24 h were immunoblotted for activated (phosphorylated, p) p-Chk1 (Ser345), p-Chk2 (Thr68), p-ATR (Ser428) or γH2AX (pH2AX at Ser139), as indicated. Total Chk1 and Chk2 protein levels independent of phosphorylation status are shown. α-Tubulin or β-actin were used as loading controls. (b) Relative expression of pChk1 and pChk2 in OE21 cells treated with equipotent (IC_50_) concentrations of cisplatin (23 μM), dppz (7 μM) or **Ru1** (21 μM) for 3 h, as determined by immunoblotting and quantified by densitometry (bottom panels). Phosphorylated protein levels relative to total protein and normalised to control are presented. γH2AX/β-actin ratio also provided. (c) Impact of **Ru1**, dppz, [Ru(phen)_2_(dppz)]^2+^ or cisplatin on viability of OE21 cells, as determined by MTT assay (24 h incubation time). Mean of quadruplicates ± S.D. and representative of two independent experiments. (d) Immunoblotting of γH2AX levels in OE21 cells after 24 h treatment with cisplatin (Cis), **Ru1** or dppz (IC_50_ and IC_70_ concentrations). [Ru(phen)_2_(dppz)]^2+^ treatment also included. β-Actin was used as a loading control. * 0.25% DMSO.

To examine DNA damage response (DDR) cell signalling activation in response to lesions generated by **Ru1**, we carried out western blot analyses of protein extracts of OE21 cells using antibodies against the phosphorylated (activated) forms of several DDR signalling proteins. In **Ru1**-treated cells, an increased level of Chk1 phosphorylation at Ser345 and phospho-ATR (at Ser428) (ATR = ataxia telangiectasia and Rad3-related protein) at 3 h onwards indicated activation of ATR/Chk1 signalling (Fig. 2a), in agreement with generation of replication stress as a result of stalled replication forks.^8^ An increased level of phospho-Chk2 (Thr68) and γH2AX (Histone H2AX phosphorylated at Ser139), both of which are generated following DNA double-strand damage,^8^ was also apparent, the level of each increased with **Ru1** exposure time (Fig. 2a). Comparable temporal DDR activation induced by **Ru1** was observed in wild type p53-containing MCF7 breast cancer cells, showing that DDR activation occurs independently of p53 status (Fig. S3†). These results show **Ru1** induces both replication stress and double-strand break (DSB) DDR pathway activation; a hallmark of replication fork collapse.^35^

### Impact of dppz on replication fork progression

Although X-ray crystal structures have confirmed that DNA intercalation of RPCs is achieved primarily through the insertion of extended coordinated polypyridyl ligand(s) between base pairs,^11^–^16^ it is unknown whether this effect drives bioactivity. As poor solubility prevented the use of free tpphz, the cellular response to free (non-coordinated) dppz (Fig. 1a) – a close structural analogue of tpphz and the prototypical polypyridyl intercalating ligand – was also examined. As seen in Fig. 1b–d, replication fork progression in OE21 cells was impaired after the addition of 7 μM dppz, as indicated by a substantial decrease in IdU tract length and large (three-fold) increase in levels of stalled forks compared to mock-treated. The similarity of the impact on replication fork progression for dppz and **Ru1** is consistent with the notion that polypyridyl ligand intercalation is responsible for replication inhibition. However, approximately two-fold greater levels of stalled forks are generated by dppz than **Ru1**, indicating a more potent replication block generated by the organic ligand (Fig. 1d). Examining DDR activation due to dppz treatment, high pChk1 levels and decreased levels of the DSB damage marker γH2AX and pChk2 activation in comparison to **Ru1** treatment were apparent, indicating reduced DSB damage pathway activation by dppz (Fig. 2b and d).

In comparison to **Ru1** and dppz, [Ru(phen)_2_(dppz)]^2+^ demonstrated substantially reduced impact on cell proliferation along with no evidence of DDR activation (Fig. 2c and d). This may be explained by the reduced cellular uptake of [Ru(phen)_2_(dppz)]^2+^ compared to **Ru1** (*vide supra*). The low bioactivity and poor nuclear targeting of [Ru(phen)_2_(dppz)]^2+^ are in agreement with other studies.^18^,^32^

### Global response to **Ru1**-induced DNA damage

**Fig. 3:**
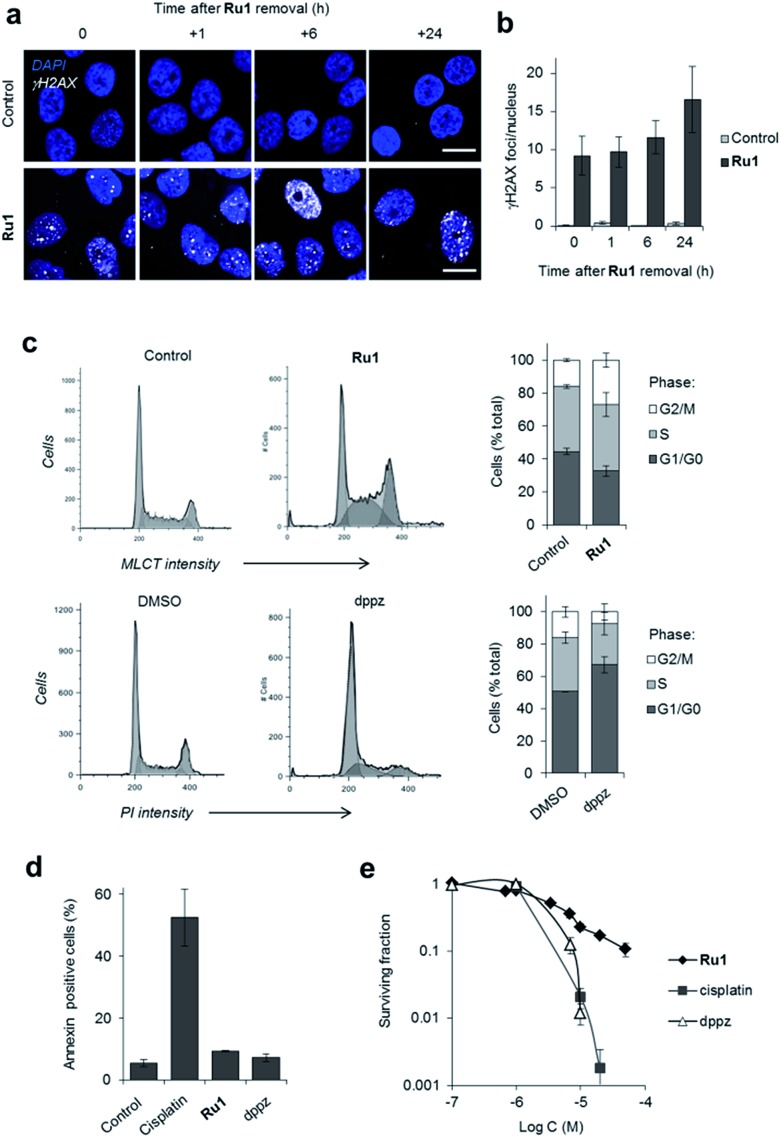
(a) Representative CLSM images of OE21 cells treated with **Ru1** (20 μM, 24 h) visualised at 0, 1, 6 and 24 h after complex removal. Immunofluorescence staining with anti-γH2AX antibody (white) provides visualisation of DSB damage. DNA (DAPI) staining included for reference. Scale bars = 20 μm. (b) Quantification of γH2AX foci/nucleus for OE21 cells treated as in (a). Data mean ± S.D. of four or five micrographs where a minimum of 20 nuclei were counted per image. (c) Impact of **Ru1** (20 μM, 24 h) or dppz (7 μM, 24 h) on cell-cycle distribution of OE21 cells, as determined by flow cytometric analysis of DNA content. DNA content in **Ru1**-treated cells was quantified using the MLCT emission of **Ru1** (see Experimental section). Data are mean of three independent experiments ± S.D. (d) AnnexinV staining of OE21 cells treated with cisplatin (23 μM), **Ru1** (21 μM) or dppz (7 μM) for 24 h, as determined by flow cytometry. Average of two independent experiments ± S.D. (e) Clonogenic survival of OE21 cells treated with **Ru1**, cisplatin or dppz (24 h incubation time). Average of triplicates ± S.D.

Depending upon the precise lesion generated, the cellular response to unrepaired DNA damage is typically either cell-cycle arrest or cell death.^36^ High levels of γH2AX foci are retained for a substantial time after **Ru1** treatment of OE21 cells (Fig. 3a and b), indicating prolonged DDR activation generated by the complex and the persistence of unrepaired DNA. Examining impact on cell-cycle progression, treatment of cycling OE21 cells with **Ru1** induced a two-fold increase in G2/M phase cells compared to control (Fig. 3c). A distinct cell-cycle response was induced in response to dppz where instead cells accumulated in G1 or early S phase (Fig. 3c). In contrast to cisplatin treatment, limited evidence of annexin-V-positive cells, no observable karyorrhexis and reduced cleaved caspase 3 expression was observed in OE21 cells treated with **Ru1** (Fig. 3d, S4 and S5†), indicating low levels of apoptosis. Despite this, a loss of proliferative capacity as a result of **Ru1** treatment was shown by clonogenic survival assay (Fig. 3e). Furthermore, numerous growth-arrested **Ru1**-treated OE21 cells appeared “flattened” with enlarged nuclei (Fig. S5†), a characteristic phenotype of senescence.^37^ These results are therefore consistent with permanent growth arrest being the primary response to **Ru1**-generated DNA damage in OE21 cells; although Trypan blue staining indicates ∼20% necrotic cell death to accompany this outcome (Fig. S5a†).

The targeting of genomic DNA without generating high levels of apoptosis by **Ru1** is a distinct cellular response compared to cisplatin. While at first this appears surprising, **Ru1** and cisplatin possess different DNA binding modalities (cisplatin forms covalent Pt–DNA adducts while **Ru1** binds non-covalently *via* intercalation) and growth arrest in response to DNA-damage is common, particularly in cells that lack functional p53 and thus possess a reduced capacity to activate apoptosis.^36^ It is also evident that **Ru1** demonstrates a different mechanism of action compared to structurally-related hydrophobic mitochondrial-targeting RPCs, which induce reactive oxygen species (ROS)-mediated apoptosis.^38^–^40^ As the hydrophilic **Ru1** instead targets nuclear DNA, this illustrates the role of organelle targeting in determining RPC bioactivity.

The differential DDR activation and cell-cycle deregulation exhibited by **Ru1** and dppz are also noteworthy. This may be rationalised by the greater levels of stalled forks generated by dppz resulting in a potent G1-S block. For **Ru1**, fork collapse instead results in slowed progression through S-phase and the accumulation of DSB damage, culminating in G2/M checkpoint activation. This indicates the Ru^II^ metal centre influences bioactivity and is not solely a “carrier” for the hydrophobic polypyridyl intercalating ligand. More detailed structural binding studies of **Ru1** and dppz with DNA could be highly illuminating and provide a molecular basis for these observations.

### **Ru1** generates metaphase chromosome non-attachment

**Fig. 4:**
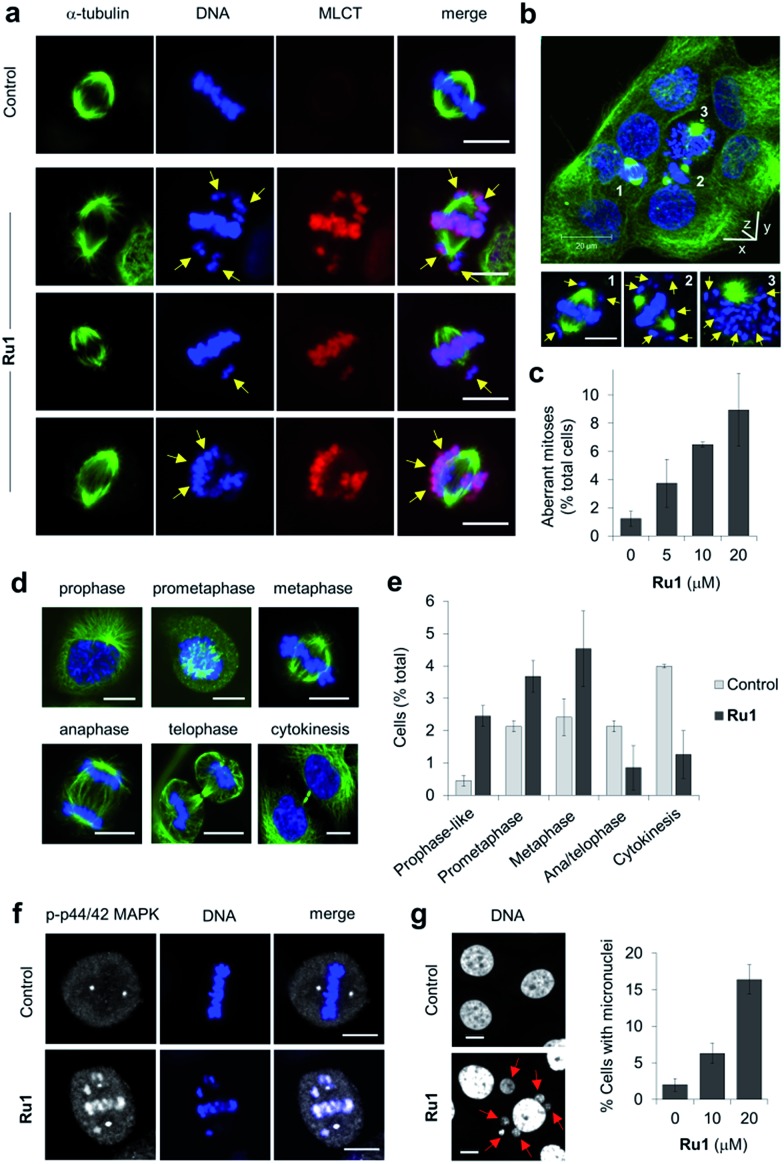
(a) Misaligned metaphase chromosomes (yellow arrows) in OE21 cells treated with **Ru1** (20 μM, 4 h). After fixation, cells were stained for α-tubulin (green) and DNA (DAPI, blue). MLCT (metal to ligand charge-transfer) emission of **Ru1** included. (b) 3D representation of **Ru1**-treated OE21 cells (20 μM, 24 h) prepared from z-stack images. Non-aligned chromosomes in labelled cells shown by yellow arrows. (c) Quantification of aberrant mitoses in **Ru1**-treated OE21 cells (24 h). Data average of two independent experiments ± S.D., >200 cells were counted for each condition. (d) CLSM images of mitotic stages of OE21 cells. (e) Quantification of mitotic sub-populations in **Ru1**-treated OE21 cells (20 μM, 24 h). Data are expressed as a percentage of total cell population. Data average of two independent experiments ± S.D., >200 cells counted per experiment. (f) Localisation of phospho(p)-p44/p42 MAP kinase in metaphase OE21 cells treated with **Ru1** (20 μM, 24 h), as determined by immunofluorescence (AlexaFluor594, white). DNA staining (DAPI, blue) included for reference. (g) Formation of micronuclei (red arrows) in OE21 cells treated with **Ru1** (20 μM, 24 h). Data average of two independent experiments ± S.D. where >100 cells were counted for each condition. Scale bars = 10 μm.

Close inspection revealed **Ru1** induced chromosome misalignment in metaphase OE21 cells (Fig. 4a), possibly as a consequence of non-attachment of sister chromatids to spindle microtubules. To test this, z-stack confocal analysis and 3D reconstruction confirmed dispersion of condensed chromosomes in **Ru1**-treated populations with evidence of complete failure of attachment to the mitotic spindle (Fig. 4b and S6, ESI Videos 1 and 2†). Misalignment was a consequence of failure of any kinetochore attachment as no monotelic, syntelic, or merotelic figures were observed. These mitotic aberrations accounted for ∼8% of the total cell population at the IC_50_ concentration of **Ru1** compared with 1.2% for control cells (Fig. 4c). Examining the impact of **Ru1** on m-phase progression, an increase in prophase, prometaphase and metaphase cells was observed with an accompanying decrease in anaphase, telophase and cytokinesis populations (Fig. 4d and e). Increased levels of chromatin-associated activated (phosphorylated) p44/42 MAPK (mitogen-activated protein kinases) of aberrant metaphases indicated activation of the spindle assembly checkpoint (SAC) by **Ru1** (Fig. 4f and S7†) and confirming the absence of merotelic attachment as the latter do not activate the SAC.^41^–^43^ In addition, treatment with **Ru1** resulted in a large increase in cells containing multiple micronuclei (Fig. 4g and S8†), indicating that despite activation of the SAC, a significant proportion of these cells fail to maintain SAC-induced mitotic arrest and progress through mitosis; such observations are a documented consequence of fragmented or detached chromosomes generated during mitosis.^44^ These results are therefore consistent with DNA damage accumulated during mitosis causing errors in chromosome segregation and spindle attachment failure, thereby resulting in SAC activation and delayed metaphase-to-anaphase transition.^45^ This phenotype has been observed with other DNA-damaging agents, but these studies have always required genetic knockdown or co-treatment with a Chk1 inhibitor to abrogate the G2 checkpoint to achieve this outcome.^46^–^48^ It is therefore significant that **Ru1** generates this effect applied as a single-agent.

### **Ru1** demonstrates distinct cancer cell-selectivity

The dual-mode mechanism of action of **Ru1** implies that rapidly-proliferating cells with an elevated mitotic index and/or mutations of mitotic checkpoint genes (characteristics of many oesophageal cancers^49^,^50^) would be more susceptible to treatment. Accordingly, a panel of p53-deficient oesophageal cancer cell lines defective in Aurora kinase function^49^,^50^ were treated with **Ru1** and relative potency assessed by MTT assay, which measures both cytotoxic and growth inhibitory (cytostatic) effects.^51^ Immortalised human small airway epithelial hSAEC1-KT cells, which are p53-functional, have a slow growth rate and low mitotic index,^52^ were used as a control. Comparable cellular and nuclear uptake of **Ru1** in the three cancer cell lines was observed (Fig. S1†), however, derived half-inhibitory (IC_50_) concentrations indicated that the relative potency of **Ru1** was greatest towards OE21 cells (Table 1 and Fig. S9†), which possess the highest mitotic index and growth rate (Table S1†). Notably, **Ru1** demonstrates a two-fold greater potency than cisplatin in OE21 cells after 72 h incubation (Table 1). Encouragingly, reduced activity of **Ru1** towards normal hSAEC1-KT cells was observed. In contrast, cisplatin showed comparable activity towards the cancer cell lines and consistent apoptosis induction across all cell lines tested, including hSAEC1-KT cells (Table 1 and Fig. S4b†). Dppz showed consistent activity towards the three cancer cell lines (Table 1) while the topoisomerase inhibitor doxorubicin demonstrated a three-fold reduction in potency towards OE21 cells compared to OE33 or FLO-1 cells after 72 h exposure (Table S2†). These results indicate the cell-selectivity profile demonstrated by **Ru1** is not a general outcome for DNA-damaging agents. The observation that dppz demonstrated comparable or greater potency than **Ru1** and substantially greater effects than [Ru(phen)_2_(dppz)]^2+^ is particularly striking as several RPC PDT candidates containing dppz-derived ligands exert bioactivity by light-activated ligand dissociation and it is often assumed their activity is due to the metal centre coordinating to DNA.^26^,^27^,^53^,^54^ These results are therefore consistent with the hypothesis that “uncaged” ligands play a significant role in these cases.^54^

### Mutagenicity of **Ru1**

DNA cross-linking agents such as cisplatin are often mutagenic.^55^ Although increased micronuclei formation indicates **Ru1** induces genotoxic stress, this effect was most apparent in OE21 cells (Table S3†), a cell line highly susceptible to genotoxic insult due to chromosomal instability and Aurora kinase B dysfunction.^49^ Employing the hypoxanthine phosphorybosyl transferase (HPRT) forward mutation assay in V79 Chinese hamster cells we show that although **Ru1** does increase mutation frequency above control, the rate is approximately two-fold lower than for cisplatin (Table 2).

**Table 2:** Mutation frequency (M. F.) induced by treatment with **Ru1** or cisplatin, as determined by HPRT-forward mutation assay in V79 Chinese hamster cells^*a*^

	M.F.^*b*^	Rel. M.F.^*c*^
Control	6.3 ± 2.4	1.0
**Ru1**	36.7 ± 7.3	5.8
Cisplatin	65.5 ± 11.3	10.4

^*a*^Cells were treated with 2 μM of each complex for 24 h.

^*b*^6-TG resistant mutations per 1 × 10^5^ viable cells.

^*c*^Ratio of induced to spontaneous mutations.

### **Ru1** radiosensitizes cancer cells *via* DNA damage enhancement

**Fig. 5:**
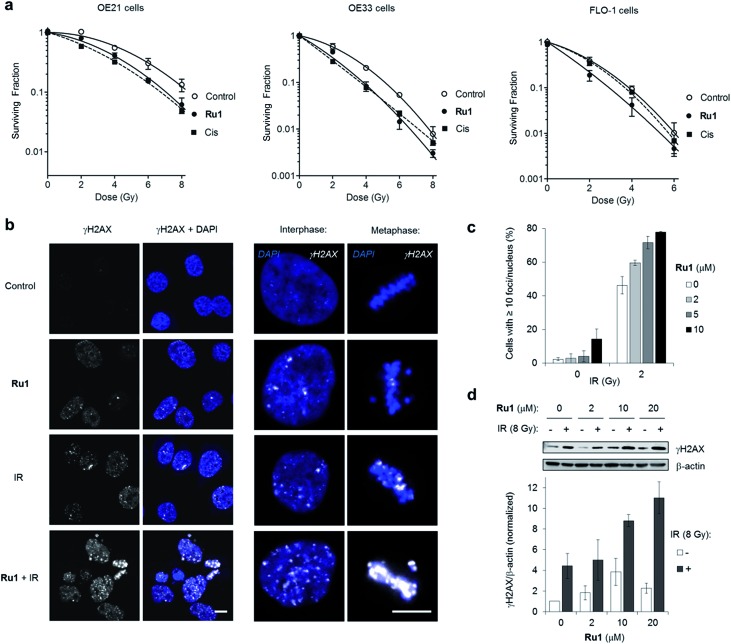
(a) Clonogenic survival of OE21, OE33 or FLO-1 cells pre-treated with **Ru1** (2 μM, 24 h) before irradiation with 0–8 Gy ^137^Cs-γ-rays. Mean ± SEM of two or three independent experiments. Data were fit to a second order polynomial function (*R*^2^ values > 0.99). Data for sub-cytotoxic doses of cisplatin (Cis) (500 nM OE21 and OE33, 300 nM FLO-1 cells) included (dashed lines). (b) CLSM images of OE21 cells treated with **Ru1** (10 μM, 24 h), IR (2 Gy), or both, where IR was applied at the end of **Ru1** treatment. Samples were fixed 1 h after irradiation. Immunofluorescence staining with anti-γH2AX antibody (white) provides visualisation of DSB damage. DNA (DAPI) staining included for reference. Scale bars = 10 μm. (c) Quantification of γH2AX foci/nucleus for OE21 cells treated as in (b). Data mean of two independent experiments ± S.D. (d) Immunoblotting (top) and corresponding densitometry (bottom) of γH2AX levels in OE21 cells treated with **Ru1** (24 h) with or without 8 Gy IR after treatment. Whole-cell extracts were prepared 1 h after irradiation and γH2AX levels were measured relative to β-actin loading controls by densitometry. Data normalised to untreated cells and are the mean ± S.D. of two independent experiments.

Many DNA-targeting drugs are employed as clinical radiosensitizers, as they induce specific DNA lesions and/or target cell-cycle progression to achieve synergy with IR targeted to cancer cells.^1^,^2^ To determine whether **Ru1** functions as a radiosensitizer, three oesophageal cancer cell lines were pre-treated with sub-IC_50_ concentrations of **Ru1** (2 μM) for 24 h before irradiation (0–8 Gy IR; ^137^Cs-γ-rays; dose rate = 0.81 Gy min^–1^) and relative cytotoxicity assessed by clonogenic survival. Fig. 5a shows that **Ru1** decreases the surviving fraction (S.F.) of all cell lines in combined treatment compared to radiation alone. Single-agent **Ru1** at this low concentration had a negligible impact on clonogenic survival (S.F. values > 0.87, Table S4†), thereby demonstrating the synergistic combination of **Ru1** with IR. Resultant dose modification factors (DMF) at surviving fractions of 0.1 were 1.19–1.31 for **Ru1**: a comparable level of radiosensitization to treatment of the same cell lines with sub-cytotoxic doses of cisplatin (DMFs at 0.1 = 1.05–1.44). The concentration required for radiosensitization by **Ru1** offers a significant improvement over previous work using the multi-intercalator [Ru(dppz)_2_(PIP)^2+^]^23^ and is similar to more cytotoxic Ru(arene)-halide monocationic complexes, which – like cisplatin – rely upon metal-centred reactivity and the formation of coordination bonds with DNA.^56^

DNA replication inhibitors often act as radiosensitizers by enhancing IR-induced cytotoxic DSB damage.^57^,^58^ Accordingly, levels of the DSB marker γH2AX^59^ in cell cultures pre-treated with **Ru1** before exposure to IR were examined. As shown in Fig. 5b and c, OE21 cells treated with **Ru1** before IR (2 Gy) demonstrated a marked increase in γH2AX foci compared to single-agent treatment groups. The large increase in γH2AX foci in concomitant **Ru1** and IR treatment indicated a substantial increase in IR-induced DSB formation compared to either treatment in isolation. A large increase in γH2AX levels is sustained even at higher doses of IR (8 Gy) and **Ru1** (10 or 20 μM) (Fig. 5d), implying a large therapeutic window exists to combine the effects of **Ru1** and IR.

Compared to work employing RPCs as photosensitizers, where *in situ* singlet oxygen or cytotoxic species formation is required for phototoxicity,^25^ radiosensitization often requires a complimentary cellular mechanism of action to enhance the DNA-damaging effects of IR.^57^,^58^ Considering many small molecules that inhibit DNA replication such as gemcitabine and 5FU (fluorouracil) are potent radiosensitizers,^58^ it seems likely that the DNA-targeting properties of **Ru1** and subsequent impact upon replication fork progression play a significant role in its radiosensitizing effects. Furthermore, G2/M phase cells are documented to be the most sensitive to IR-induced DNA damage,^1^,^2^ and so the G2 arrest and metaphase block generated in response to **Ru1** – induced DNA damage would also be predicted to increase radiosensitivity; a concept supported by the high levels of γH2AX visible in **Ru1**-treated metaphase cells exposed to IR (Fig. 5b). In addition to these effects, work employing cisplatin has also indicated DNA repair inhibition contributes to radiosenstization,^4^ and it will be interesting to measure DNA repair kinetics in **Ru1**-radiosensitized cells. Finally, although this work has established DNA as the primary target of **Ru1**, the peripheral coordination site on the tpphz ligand may additionally chelate metal ions.^60^,^61^ In agreement with this principle, the addition of ten-fold excess Zn^2+^ and Fe^2+^ ions to free or DNA-bound **Ru1** resulted in a clear decrease in MLCT emission of the complex (Fig. S10†). Moreover, this effect was reversed by the addition of the metal chelator EDTA (ethylenediaminetetraacetic acid) for Zn^2+^ binding but, interestingly, not Fe^2+^, thereby demonstrating a high affinity of **Ru1** for ferrous iron. These observations raise the possibility that **Ru1** functions as a metal ion chelator in addition to binding DNA. While the possibility of intracellular chelation impacting bioactivity cannot be discounted, this effect would not be predicted to interfere with intercalative DNA binding^60^,^61^ and the rapid block of replication fork progression by **Ru1** and DDR activation timeframe are consistent with results obtained using non-chelating DNA-binding compounds such as [Ru(dppz)_2_(PIP)^2+^].^23^,^62^ However, this may be of relevance to other effects caused by **Ru1** as iron chelators have been indicated to demonstrate radiosensitizing properties.^63^,^64^ Future work will explore these concepts.

## Conclusions

In summary, we present a detailed characterisation of the cellular response to [Ru(phen)_2_(tpphz)]^2+^ (**Ru1**) in p53-deficient oesophageal cancer cells, finding this ruthenium(ii) metallo-intercalator induces a potent replication block accompanied by replication stress and DSB damage repair pathway activation, without triggering apoptosis. To our knowledge, this is the first example of a substitutionally inert ruthenium(ii) mono-intercalator demonstrated to function as a replication inhibitor. In parallel to this, metaphase chromosome attachment is impaired by **Ru1**. This multi-mode mechanism of action results in growth inhibition of highly proliferative oesophageal cancer cells with elevated mitotic indices. Finally, efficient radiosensitization through synergistic DNA damage enhancement illustrates the efficacy of **Ru1** in combination with IR, where the lower mutagenicity and reduced cytotoxicity of **Ru1** compared to cisplatin would be predicted to be advantageous in its use alongside radiotherapy.

## Conflicts of interest

There are no conflicts of interest to declare.

## Supplementary Material

Supplementary movie

Supplementary movie

Supplementary information
