# Supplementary material for: A three-in-one-bullet for oesophageal cancer: replication fork collapse, spindle attachment failure and enhanced radiosensitivity generated by a ruthenium(ii) metallo-intercalator
Source: Chem Sci. 2017 Nov 16;9(4):841–9. doi: 10.1039/c7sc03712k (PMC5870190; doi:10.1039/c7sc03712k)
Supplement: Supplementary file 3 [file SC-009-C7SC03712K-s003.pdf]

Electronic Supplementary Material (ESI) for Chemical Science.
This journal is © The Royal Society of Chemistry 2017

Supplementary Information for:

A three-in-one-bullet for oesophageal cancer: Replication fork collapse, spindle attachment failure and enhanced radiosensitivity generated by a ruthenium(II) metallo-intercalator†

Martin R. Gill,*[a] Paul J. Jarman,[b,c] Swagata Halder,[a] Michael G. Walker,[b] Hiwa K. Saeed,[b] Jim A. Thomas,[b] Carl Smythe,[c] Kristijan Ramadan,[a] Katherine A. Vallis*[a]

a CRUK/MRC Oxford Institute for Radiation Oncology, Department of Oncology, University of Oxford, Oxford, UK. Email: martin.gill@oncology.ox.ac.uk; katherine.vallis@oncology.ox.ac.uk

b Department of Chemistry, University of Sheffield, Sheffield, UK

c Department of Biomedical Science, University of Sheffield, Sheffield, UK

Contents

Experimental… p1

Supplementary Tables… p4

Supplementary Schemes and Figures… p6

Supplementary References… p17

Experimental

Chemicals, antibodies and cell lines. **Ru1** was prepared as described by Bolger, *et al.*,[1] and converted to the dichloride salt by anion metathesis. Dppz was prepared as described by Friedman, *et al.*[2] [Ru(phen)$_2$(dppz)]Cl$_2$ was prepared based on the synthetic scheme described by Hartshorn and Barton.[3] All NMR, mass spec. and elemental analysis were in agreement with published data. Unless stated otherwise, all other chemicals were obtained from Sigma. Note: The results within this study indicate **Ru1** is a potential mutagen and should therefore be handled with caution. Antibodies: p-Chk1 (Ser345), p-Chk2 (Thr68), cleaved caspase 3, p-ATR (Ser428), p-BRCA1 (Ser1524) (all Cell Signaling), γH2AX (Millipore), Chk1 (Santa Cruz), Chk2, α-tubulin (both Abcam) and β-actin (Sigma). Phospho-p44/42 MAPK (Erk1/2) (Thr202/Tyr204) (Cell Signaling) was a generous gift from Dr A. Azad. AlexaFluor488 and AlexaFluor594-conjugated anti-mouse or anti-rabbit secondary antibodies were from Cell Signaling. OE21 Human oesophageal squamous cell carcinoma, OE33 Human oesophageal carcinoma and FLO-1 Human Distal oesophageal adenocarcinoma cell lines were a generous gift from E. Hammond. HSAEC1-KT human small airway epithelial cells were purchased from ATCC.

Cell culture. OE21 and OE33 cells were cultured in RPMI supplemented with 10% FBS and penicillin/streptomycin. FLO-1 cells were cultured in DMEM supplemented with 10% FBS and penicillin/streptomycin. HSAEC1-KT cells were cultured in Small Airway Epithelial Cell Growth Medium (Lonza) supplemented with the contents of the SAGM SingleQuot Kit. Cell lines were maintained at 37 °C in an atmosphere of 5% CO_2 and sub-cultured by Trypsin. Cell lines were used at passage numbers 40 or lower and checked to be mycoplasma-free on a monthly basis. Stock solutions of **Ru1** (2 mM), cisplatin (2 mM) were prepared in PBS before dilution in cell media. Dppz (4 mM) was prepared in DMSO and diluted in cell media immediately to minimise compound degradation. Cells treated with **Ru1** were shielded from light to minimise any

phototoxic contribution to bio-activity. Doxorubicin (Sigma) was prepared in doubly-distilled water (10 mg/mL, 17.4 mM). $[Ru(phen)_2(dppz)]^{2+}$ (2 mM) was prepared in doubly-distilled water.

Sub-cellular fractionation. OE21 or OE33 cells were treated in 12 well plates, washed with cold PBS (2x2 mls) and nuclear and cytosol fractions obtained using the Nuclei EZ Lysis kit (Sigma). Briefly, cells were washed with acidified PBS (pH 2.5) to remove the membrane-bound fraction and 0.4 ml EZ lysis buffer was added. Cells were detached by scraping, collected into eppendorfs, vortexed briefly and left for 5 minutes on ice. Samples were centrifuged (500g, 5 mins) and the supernatant (cytosol fraction) aspirated. The pellet (nuclear fraction) was re-suspended in 200 μl RIPA buffer. Fractionation of compartments was verified by immunblotting using anti-α-tubulin (Sigma) and anti-histone H2AZ (Abcam) for cytosol and nuclear fractions, respectively. Ruthenium content was determined by ICP-MS analysis as described in a recent publication.[4]

DNA fibre assay. The DNA fibre assay was performed as described previously.[5] Briefly, OE21 cells were labelled with 30 μM CldU for 30 mins and then 250 μM IdU for an additional 30 mins. Either **Ru1** (21 μM) or dppz (7 μM) was added during the second (IdU) nucleotide step (30 mins). DNA replication was terminated using ice-cold PBS and cells lysed (200 mM Tris-HCl pH 7.4, 50 mM EDTA and 0.5% SDS). DNA fibres were spread onto glass slides, fixed with methanol:acetic acid (3:1) and denatured with 2.5 M HCl. After blocking with 2% BSA, fibres were stained with anti-rat and anti-mouse 5-bromo-2'-deoxyuridine (BrdU) that specifically recognise either CldU (Abcam, dilution 1:500) or IdU (BD Sciences, 1:100). Anti-rat Cy3 (Jackson Immuno Research, 1:300) and anti-mouse Alexa-488 (Molecular Probes, 1:300) were used as the respective secondary antibodies. CldU- and IdU-labelled tracts were visualised using a Leica DMRB microscope with a DFC360FX camera and tract length measured by ImageJ software. Statistical analysis was performed by GraphPad Prism software using an unpaired t-test.

Western blotting. Treated samples were washed with cold PBS and lysed in RIPA (radioimmunoprecipitation assay) buffer containing protease inhibitors (10 µg/mL leupeptin, 2 µg/mL pepstatin, 50 µg/mL antipain, 2 µg/mL aprotinin, 20 µg/mL chyprostatin, 2 µg/mL benzamidine, 1 mM phenylmethanesulfonyl fluoride) and phosphatase inhibitors (50 mM NaF, 1 mM Na_3VO_4 and 20 mM β-glycerophosphate). Protein content was determined by BCA assay. Aliquots of cell lysates (10-50 µg total protein) were prepared in standard Laemmli buffer, heated at 95 °C for 5 minutes and resolved by NuPAGE® 4–12% Bis-Tris gels and LDS-PAGE. Gels were transferred onto nitrocellulose membrane and probed with primary antibodies in 5% BSA (bovine serum albumin) solutions at the following dilutions: 1/1000 dilution for majority of Cell Signaling antibodies and γH2AX. 1/500 for anti-p-ATR and anti-cleaved caspase 3. 1/200 anti-Chk1. 1/5000-1/10,000 anti- α-tubulin or β-actin. Reactions were visualised with a suitable secondary antibody conjugated with horseradish peroxidase (1/5000 dilution, Thermo). WesternSurePREMIUM (Li-Cor) chemiluminescent substrates with X-ray development (Fuji medical film and Optimax 2010 processor) or digital analysis (LiCor C-Digit Blot Scanner) were used to visualise protein expression.

AnnexinV staining. FITC Annexin V/Dead Cell Apoptosis Kit (Thermo) was used according to the manufacturer's instructions. Briefly, OE21 cells were incubated with **Ru1**, dppz or cisplatin in 6 well plates at 24 h IC_{50} concentrations for 24 h, harvested via Trypsin/centrifugation and washed with cold PBS. Approximately 1 x 10^5 cells were re-suspended in 100 μl binding buffer and 5 μl Annexin V and 5 μl PI added. This was incubated for 15 mins at room temperature, 400 μl binding buffer added and analysed by flow cytometry (FL1 and FL3 channels for FITC and PI respectively). A minimum of 10,000 cells were counted per condition.

Cell-cycle analysis. Cells were washed with PBS and detached using Trypsin. After centrifugation (1000 rpm, 5 min) cells were fixed in cold 70% ethanol for 30 mins, centrifuged (1000 rpm, 5 mins) and re-suspended in PBS. Although using

propidium iodide (PI) is standard to quantify DNA content using flow cytometry, bleed-through of the MLCT emission of internalised **Ru1** into the FL2 channel resulted in false "sub-G1" peaks. To remove these artefacts, a higher concentration of **Ru1** (100 μM, 10 mins) was additionally applied *post*-incubation and fixation to treated and control samples, acting as a substitute DNA dye instead of PI. After staining, cells were centrifuged (1000 rpm, 5 mins) and re-suspended in PBS. Samples were analysed using a Biosciences LSRII Flow Cytometer using the FL2 channel to collect (recognisable) cell-cycle profiles. This generated a cell-cycle profile comparable to PI staining in untreated cells (see Figure S11). A minimum of 10,000 cells were counted for each sample and data were processed using FloJo software.

Microscopy and immunofluorescence. Cells were seeded on ibidi 35 mm μ-dishes (Thistle Scientific), allowed to adhere for 24 h and treated as stated in the main text. Cell media was removed, cells washed with PBS and fixed with formaldehyde (4%, 10 mins). Cells were permeablised with Triton (0.5% in PBS, 5 mins) and washed with PBS. Samples were blocked with BSA (3% in PBS-T) for 1 h before incubation with primary antibody (either anti-α-tubulin or anti-γH2AX, 1/500 dilution) in BSA (3% in PBS-T) for 1 h. Samples were washed 3x in PBS-T and incubated with Alexa Fluor-conjugated secondary antibodies (3% in PBS-T, 1 h, 1/250 dilution). After further washing (3x5 min PBS-T) samples were co-stained with DAPI (5 ng/ml, 2 min), fresh PBS added and visualised by confocal microscopy. Samples were visualised using a Zeiss LSM 780 or LSM 710 inverted confocal microscope and EC Plan-Neofluar 40x/1.30 Oil objectives. DAPI (λ_{ex} = 405 nm) and AlexaFluor594 (λ_{ex} = 594 nm) were collected at 410-495 nm and 606-699 nm respectively. AlexaFluor488 (λ_{ex} = 488 nm) emission was collected from 500-530 nm to prevent spectral overlap with **Ru1** emission. γH2AX foci were counted using ImageJ software.

MTT assay. OE21, FLO-1 or hSAEC1-KT cells were seeded in 96 well plates at 20,000 cells/well (for 24 h treatment) or 10,000 cells/well (for 72 h treatment) and allowed to adhere for 24 h before treatment. OE33 cells were seeded in 48 well plates at 40,000 cells/well (24 h treatment) or 20,000 cells/well (72 h treatment). Cancer cell lines were treated with chemicals dissolved in RPMI media for consistency. After incubation, 0.5 mg/ml MTT (thiazolyl blue tetrazolium bromide) dissolved in serum-free medium was added for 60 minutes and the formazan product eluted using acidified isopropanol. The absorbance at 540 nm was quantified by plate reader (reference wavelength 650 nm). The metabolic activity of the cell population was determined as a percentage of a negative (solvent) control.

Trypan Blue exclusion assay. OE21 cells were seeded in 6 well plates at 40,000 cells/well and allowed to adhere for 24 h. Cell cultures were treated as stated, media containing detached/dead cells were removed and retained, and adherent collected by Trypsin and centrifugation. Adherent and detached cells for each sample were combined, concentrated via centrifugation and re-suspended in 0.5 ml PBS containing 0.04% Trypan Blue solution. Trypan Blue negative and Trypan Blue positive cells were counted by haemocytometer. A minimum of 200 cells were counted for each sample.

HPRT (hypoxanthine-guanine phosphoribosyl transferase) forward mutation assay. V79 Chinese hamster cells were plated at $1x10^6$ cells/100 mm dish in regular culture medium (DMEM supplemented with 10% FBS, 100 mg ml-1 streptomycin, 100 units ml-1 penicillin, and 2 mM glutamine at 37 °C in a humidified atmosphere containing 5% CO_2). After a 24 hr growth period, the chemical agent was added to the medium and the cultures were incubated for 24 hr. The cultures were washed with PBS, cells were detached by trypsin (0.05%) and cell number was determined. For the determination of cloning efficiency, from the untreated and each treated condition, 500 cells were plated in a 100 mm dish in regular medium. After 7 days' incubation, the resulting colonies were stained with methylene blue and those containing 100 or more cells were counted. Cloning efficiency was determined and is usually expressed in percent relative to the untreated control culture (which usually has 80% or better efficiency). For the determination of mutation induction, from the untreated and each treated condition, $2x10^5$ cells were plated in 10 x 100 mm dishes in selective media (regular media

+ 5 μg/ml 6-thioguanine (6TG)). After incubation for 10 days, colonies were stained with methylene blue and those containing 100 or more cells were counted. The mutation frequency is determined by dividing the total number of mutant colonies in the 6TG medium by the total number of cells plated, corrected by the cloning efficiency. Cloning efficiency = colonies on cloning plates/500. Mutation frequency = 6TG resistant colonies/number of cells plated on selection x cloning efficiency.

Clonogenic survival assay. OE21, OE33 or FLO1 cells were treated with 2 μM **Ru1** for 24 h before irradiation with 0-8 Gy using a ^{137}Cs γ-irradiator (IBL637, CIS Bio Int.; dose rate = 0.809 Gy min^{-1}). 1 h after irradiation, cells were detached using Trypsin and re-seeded in 6 well plates at a density of 300 – 20,000 cells/well in triplicate in an incrementally greater number of cells with increasing IR dose to ensure adequate colony formation. Cells were incubated for 7–14 days after re-seeding to allow colony formation before being fixed with 10% methanol, 10% acetic acid and stained with 0.4% methylene blue. OE33 cells required an additional formaldehyde fixation (4% in PBS, 5 mins) step before the addition of methylene blue solution or colony detachment occurred. Colonies containing 50 cells or greater were counted using a Gelcount instrument and accompanying software (Oxford Optronix). Plating efficiencies were determined for each treatment condition and normalised to an untreated control to provide the surviving fraction (S. F.). S.F. versus radiation dose curves were plotted in GraphPad Prism and fit using a second order polynomial function (R^2 values > 0.99). Dose modifying factors (DMF) at a SF=0.1 were calculated using the equation: Dose modifying factor (DMF) = IR dose to give S.F. 0.1/IR dose plus compound to give S.F.0.1.

Supplementary Tables

Table S1. Mitotic indices of OE21, OE33 and FLO1 cell lines. The mitotic index of OE21 and FLO-1 cells was determined by immunofluorescence and DAPI staining and is the average of two experiments.

Cell line	Doubling time (hrs)	Mitotic index (%)	References
OE21	29	7.1 ± 1.1	6, 7, this work
OE33	33	3.4 ± 2.2	6, 7
FLO-1	40	4.5 ± 0.3	8 , this work
hSAEC1-KT	>50	1.0 ± 0.0	6, 7,9

Table S2. IC50 values (μM) of doxorubicin (DOX) towards OE21 oesophageal squamous cell carcinoma, OE33 and FLO-1 human oesophageal adenocarcinoma cancer cell lines, as determined by MTT assay. Data mean of two independent experiments +/− S.D (24 or 72 h constant incubation).

	OE21		OE33		FLO1	
	24 h	72 h	24 h	72 h	24 h	72 h
DOX	4.2 ± 0.3	0.21 ± 0.07	8.1 ± 2.7	0.07 ± 0.03	6.1 ± 1.9	0.05 ± 0.02

Table S3. Micronuclei formation in OE21, OE33, FLO-1 or MCF7 cells treated with **Ru1** (24 h incubation).

Cell line	Treatment	Cell number	Cells with micronuclei	Percentage
OE21 (n=1)	Control	154	4	2.6
	20 μM **Ru1**	129	23	17.8
OE21 (n=2)	Control	146	2	1.4
	20 μM **Ru1**	107	16	15.0
OE33	Control	181	3	1.7
	45 μM **Ru1**	177	6	3.4
FLO-1	Control	156	5	3.2
	44 μM **Ru1**	152	5	3.3
MCF7	Control	171	9	5.3
	40 μM **Ru1**	210	13	6.2

Table S4. Surviving fraction (S.F.) data of oesophageal cancer cells pre-treated with either **Ru1** or cisplatin (Cis) before irradiation with 0, 2, 4, 6 or 8 Gy ^{137}Cs-γ-rays, as determined by clonogenic survival assay. Mean +/− S.D. of two or three independent experiments. Data were normalised to a non-complex and non-IR treated control for each experiment. IR = 0 Gy conditions correspond to complex treatment without IR. N.D. = not done.

Treatment	IR (Gy)	Cell line					
		OE21		OE33		FLO-1	
		S.F.	S.D.	S.F.	S.D.	S.F.	S.D.
Control (blank)	0	1.000	0.054	1.000	0.054	1.000	0.011
	2	1.028	0.106	0.584	0.126	0.384	0.138
	4	0.552	0.028	0.204	0.001	0.093	0.027
	6	0.304	0.091	0.054	0.004	0.010	0.012
	8	0.133	0.045	0.008	0.005	N.D.	N.D.
**Ru1**[a]	0	1.062	0.088	0.956	0.023	0.873	0.147
	2	0.801	0.028	0.447	0.003	0.188	0.084
	4	0.410	0.060	0.083	0.027	0.042	0.031
	6	0.152	0.012	0.014	0.006	0.005	0.003
	8	0.062	0.025	0.003	0.001	N.D.	N.D.
Cis[b]	0	1.000	0.122	0.993	0.100	0.929	0.111
	2	0.587	0.032	0.280	0.028	0.340	0.008
	4	0.320	0.009	0.077	0.019	0.080	0.005
	6	0.159	0.019	0.022	0.010	0.007	0.000
	8	0.049	0.008	0.005	0.004	N.D.	N.D.

[a] 2 μM **Ru1** for 24 h before IR treatment. [b] 500 nM Cis in OE21 and OE33 cells, 300 nM Cis in FLO-1 cells for 24 h before IR (higher concentrations of cisplatin impacted colony survival).

Supplementary Schemes and Figures

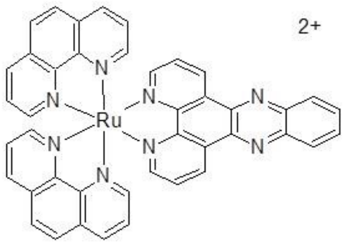

Scheme S1. Structure of $[Ru(phen)_2(dppz)]^{2+}$.

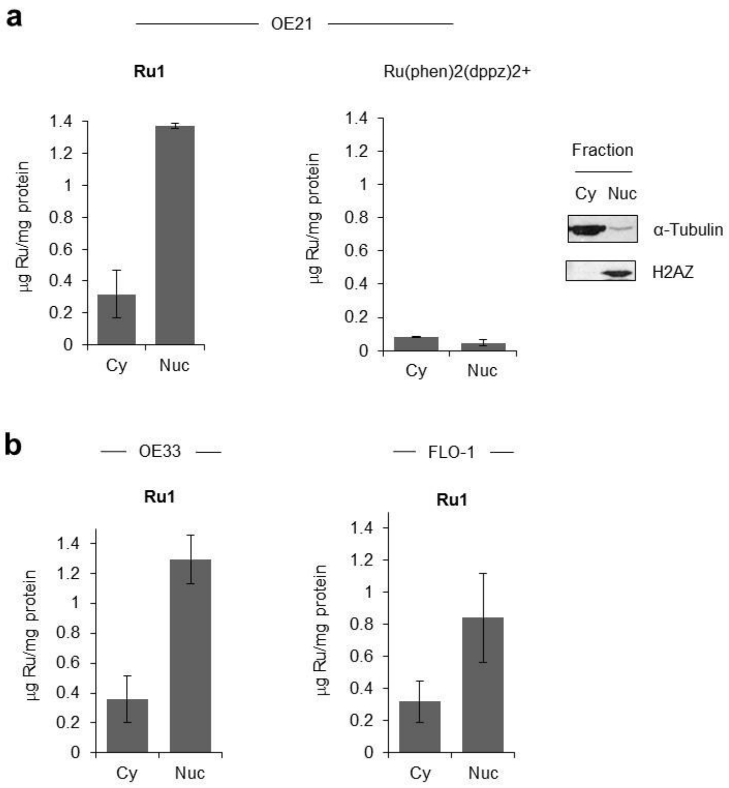

Figure S1. (a) Ruthenium content of OE21 oesophageal squamous cell carcinoma cells incubated with either **Ru1** or $[Ru(phen)_2(dppz)]^{2+}$ (20 µM, 24 h). Isolated cytosol (Cy) and nuclear (Nuc) fractions were obtained and Ru content determined by ICP-MS. Data expressed as µg Ru per mg of cell protein (as determined by BCA assay). Mean of two independent experiments +/- S.D for **Ru1** and mean of triplicates +/- for $[Ru(phen)_2(dppz)]^{2+}$. Successful fractionation of cytosol and nuclei was confirmed by immunoblotting using α-tubulin and H2AZ antibodies for cytosol- and nuclei-enriched fractions respectively (right). (b) Sub-cellular ruthenium content of OE33 or FLO-1 oesophageal adenocarcinoma cells incubated with **Ru1** (20 µM, 24 h), and processed as described for (a). Mean of two independent experiments +/- S.D.

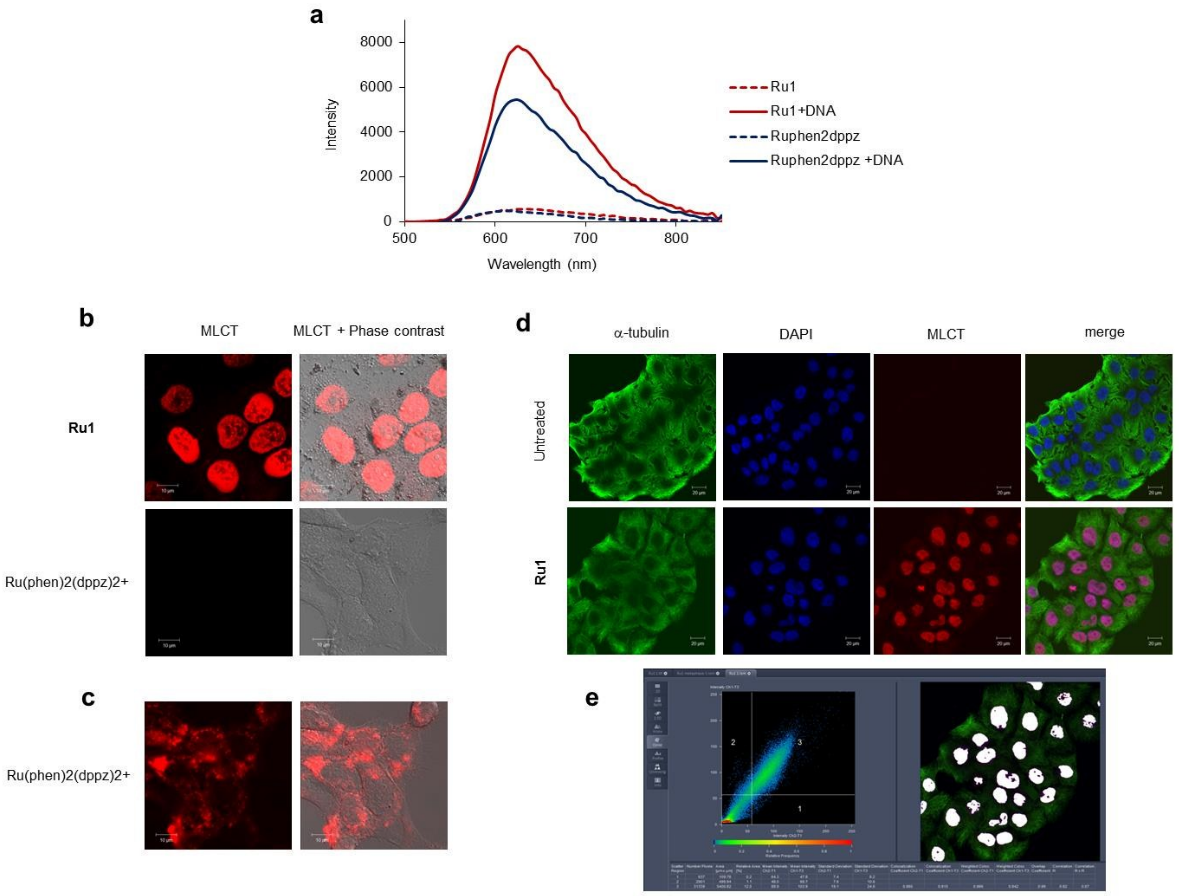

Figure S2. (a) MLCT emission spectra of **Ru1** (2 μM) or $[Ru(phen)_2(dppz)]^{2+}$ (10 μM) with the addition of 2 ng/ml calf thymus DNA (5 mM Tris buffer pH 7.4). Spectra were collected using the same instrumental conditions (λ_{ex} = 458 nm). (b) Live cell CLSM imaging of OE21 cells treated with $[Ru(phen)_2(dppz)]^{2+}$ or **Ru1** (100 μM, 3 hrs, serum-free media) showing intracellular MLCT emission. Identical microscopy settings were employed for MLCT acquisition. (c) Cells treated with $[Ru(phen)_2(dppz)]^{2+}$ (b, lower panels) visualised with increased laser power and detector gain. (d) CLSM of OE21 cells treated with **Ru1** (20 μM, 4 h) showing MLCT emission. After fixation, cells were stained for α-tubulin (green) and DNA (DAPI, blue). (e) Co-localisation of MLCT (T1 channel, x-axis) and DAPI (T3 channel, y-axis) signals. Area of overlap shown in white (right). (Screenshot from ZEN imaging software).

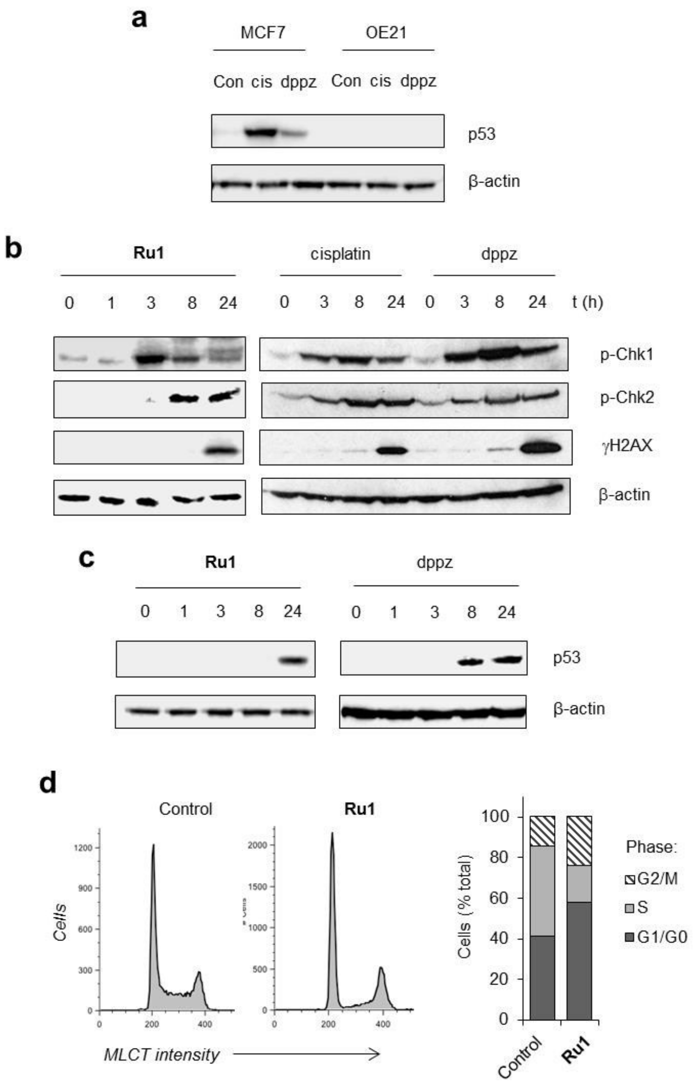

Figure S3. (a) Whole-cell extracts of MCF-7 or OE21 cells treated with cisplatin (17 µM) or dppz (8 µM) for 24 h and immunoblotted for p53. β-actin was used as a loading control. (b) Whole-cell extracts of MCF-7 cells treated with **Ru1** (40 µM), cisplatin (17 µM) or dppz (8 µM) for 1, 3, 8 or 24 h (as indicated) were immunoblotted for activated (phosphorylated, p) DDR proteins p-Chk1 (Ser345), p-Chk2 (Thr68) or γH2AX. β-actin was used as a loading control. (c) Whole-cell extracts of MCF-7 cells treated with **Ru1** (40 µM) or dppz (8 µM) for 1, 3, 8 or 24 h (as indicated) were immunoblotted for p53 expression. β-actin was used as a loading control. (d) Impact of **Ru1** (40 µM, 24 h) on cell-cycle distribution of MCF-7 cells. DNA content was determined using the MLCT emission of **Ru1** (100 µM applied post-treatment, see Materials section) and analysed by flow cytometry.

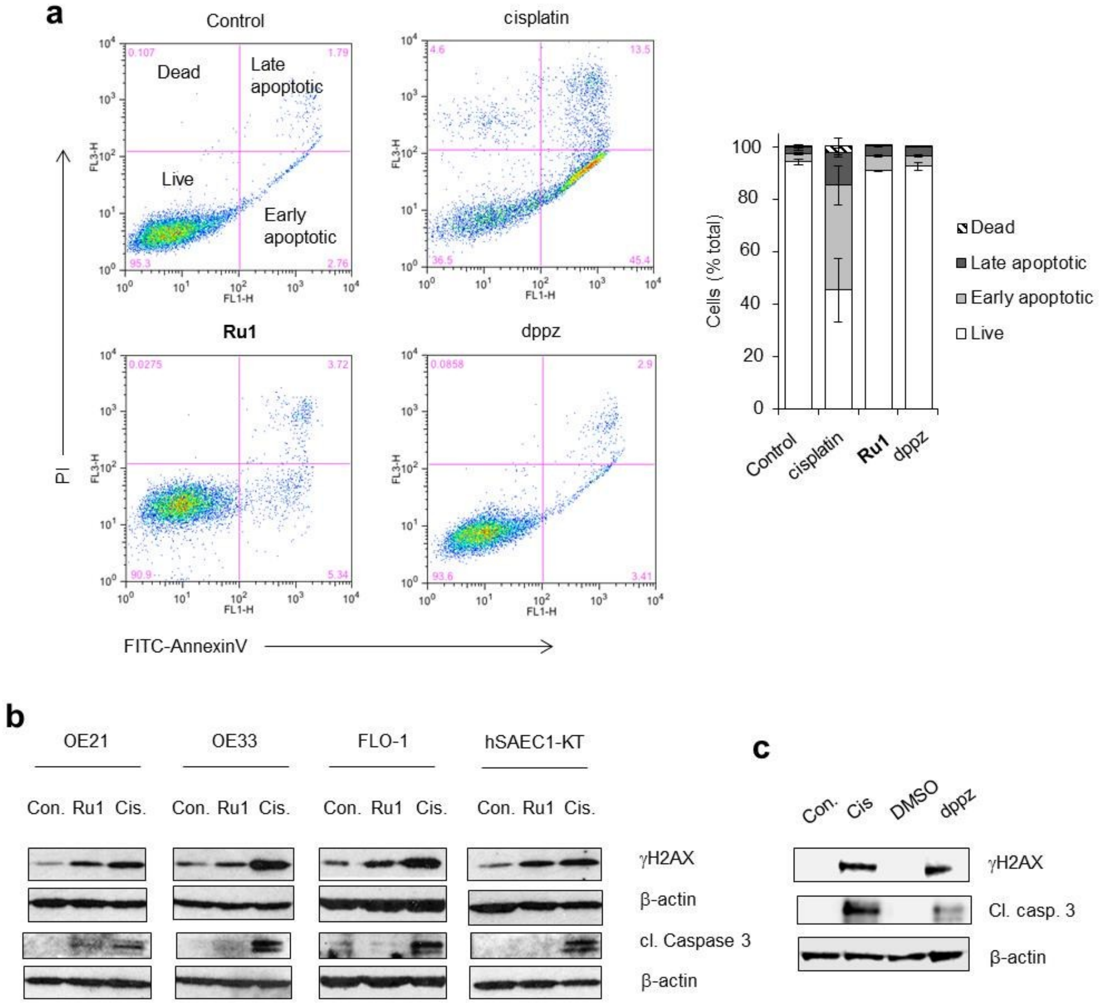

Figure S4. (a) AnnexinV/PI staining of OE21 cells treated with cisplatin (23 μM), **Ru1** (21 μM) or dppz (7 μM) for 24 h, as determined by flow cytometry. Quantification of early/late apoptotic cells (right). Average of two independent experiments +/- S.D. A minimum of 10,000 cells were counted per condition. (b) Expression of DNA damage (indicated by γH2AX levels) and apoptosis marker cleaved caspase-3 in cells treated with cisplatin or **Ru1** (20 μM, 24 h), as determined by immunoblotting. β-actin was used as a loading control. (c) Generation of DSB marker γH2AX and cleaved caspase-3 in OE21 cells treated with cisplatin or dppz (10 μM) for 24 h, as determined by immunoblotting. β-actin was used as a loading control.

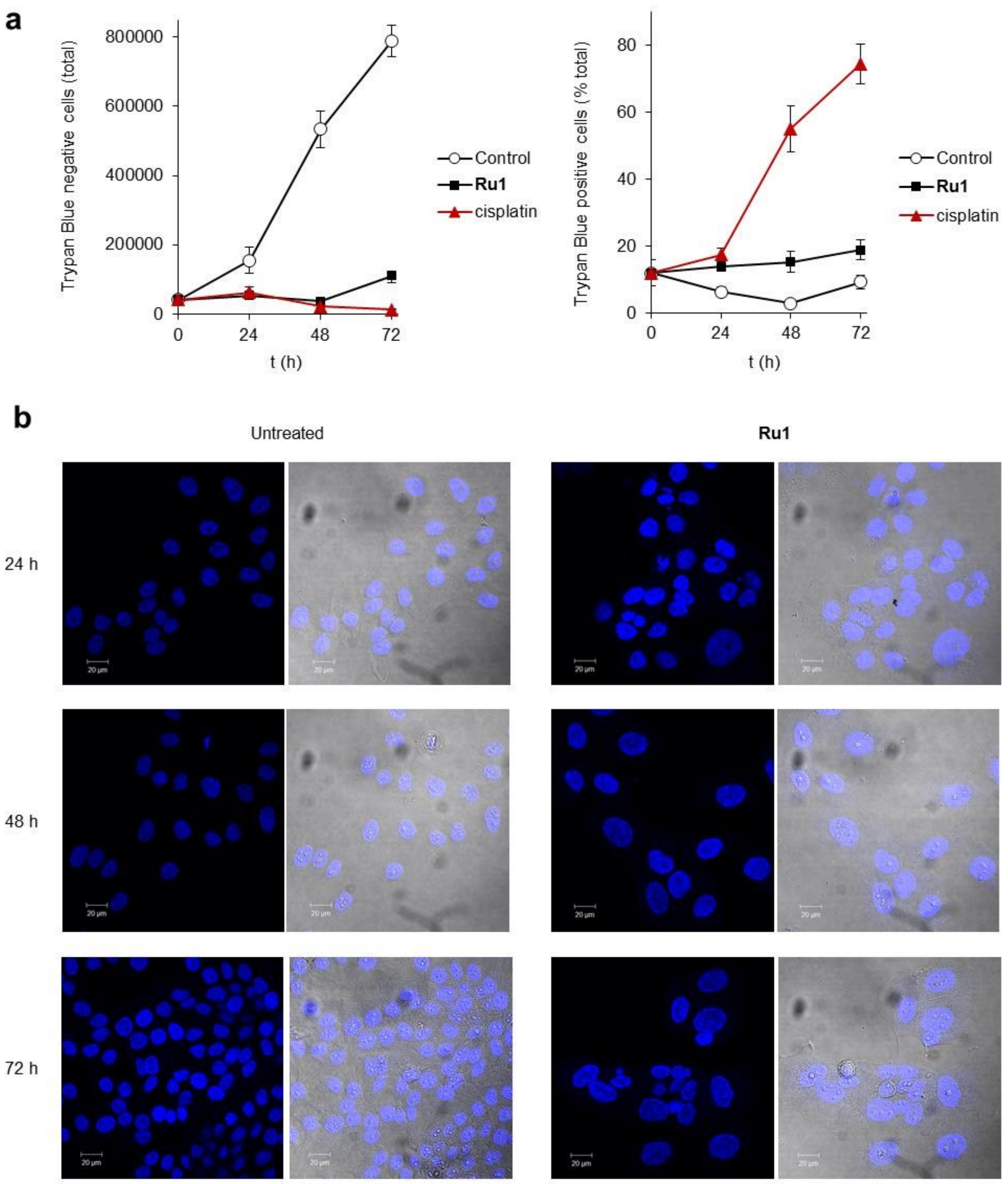

Figure S5. (a) Number of viable cells (Trypan Blue negative, left) after treatment with **Ru1** (21 μM) or cisplatin (23 μM) for 24, 48 or 72 h constant exposure (in triplicate, +/- S.D.). Trypan Blue positive staining (i.e. non-viable cells) are included as percentage of total cells counted (right). (b) CLSM images of OE21 cells either untreated or treated with **Ru1** (10 μM) for 24, 48 or 72 h constant exposure. Note the appearance of large, "flattened" cells with enlarged nuclei as a result of **Ru1** treatment (bottom right images). DAPI (blue) and phase contrast images shown.

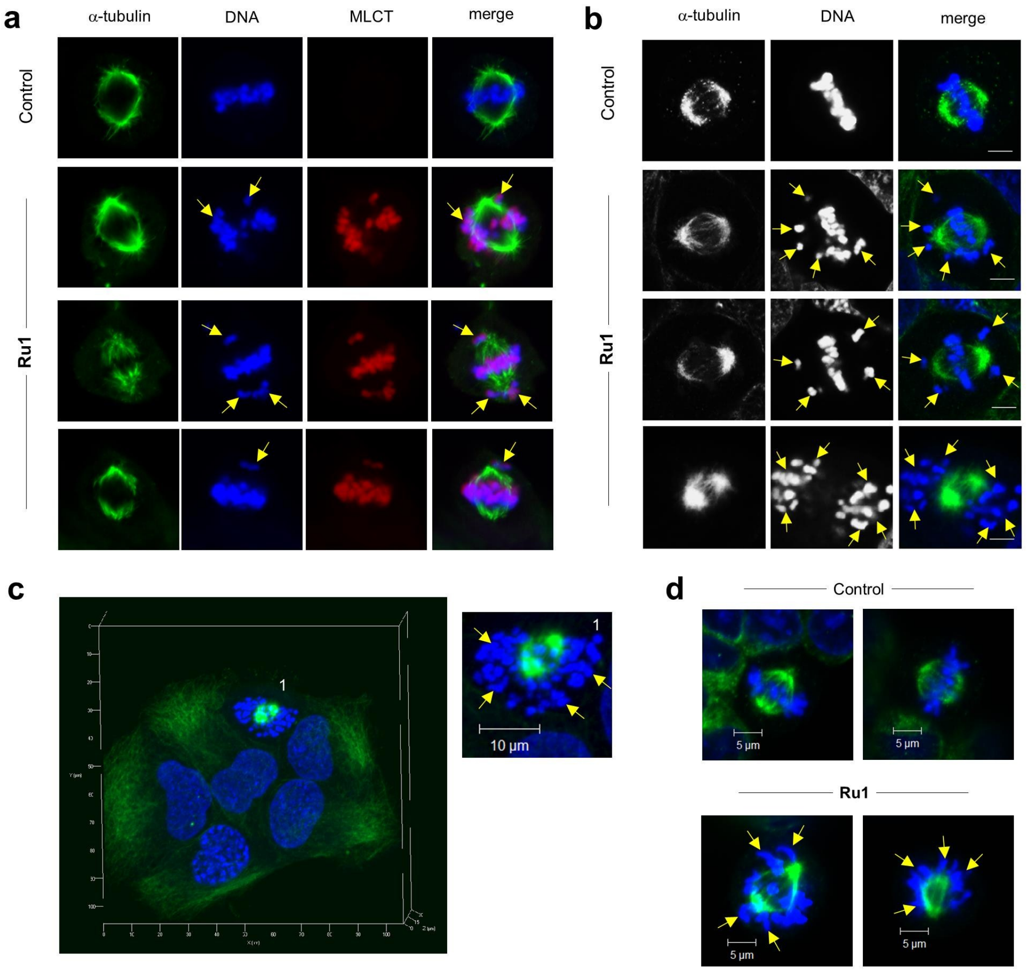

Figure S6. (a) OE21 cells treated with **Ru1** (20 μM, 4 h) stained for α-tubulin (immunofluorescence, green) and DNA (DAPI, blue). Misaligned chromosomes are indicated by arrows. MLCT emission of **Ru1** included for reference. (b) Examples of misaligned metaphase chromosomes induced by treatment of OE21 cells with **Ru1** (20 μM, 24 h). α-tubulin (immunofluorescence, green) and DNA (DAPI) staining shown. Scale bars = 5 μm. (c) 3D representation of **Ru1**-treated OE21 cells prepared from z-stack images. (d) FLO-1 cells treated with **Ru1** (44 μM, 24 h) stained for α-tubulin (immunofluorescence, green) and DNA (DAPI, blue) also demonstrate misaligned metaphase chromosomes (bottom panels).

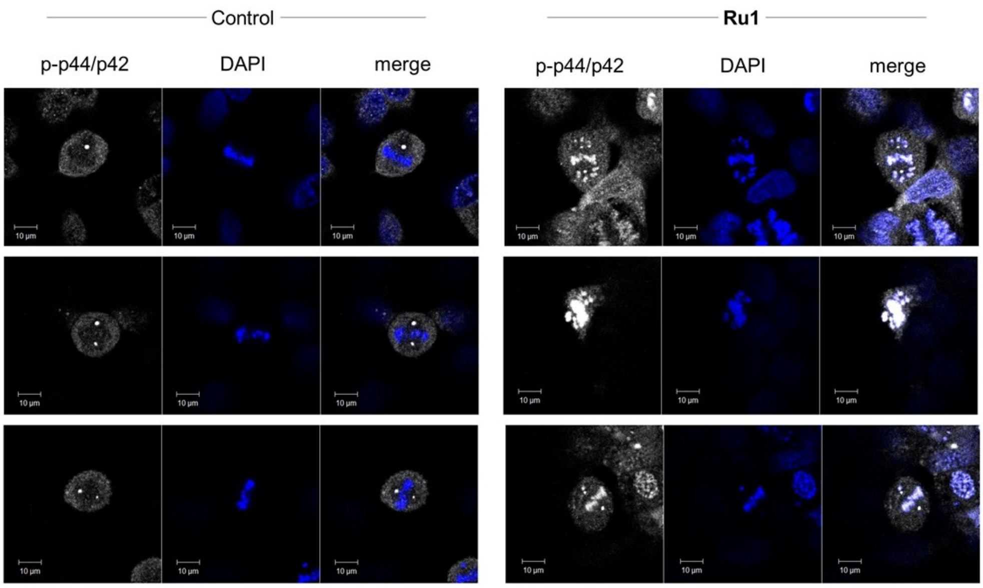

Figure S7. Localisation of phospho(p)-p44/p42 MAP kinase in metaphase OE21 cells treated with **Ru1** (20 μM, 24 h), as determined by immunofluorescence (Alexa Fluor 594, white). DNA staining (DAPI, blue) included for reference.

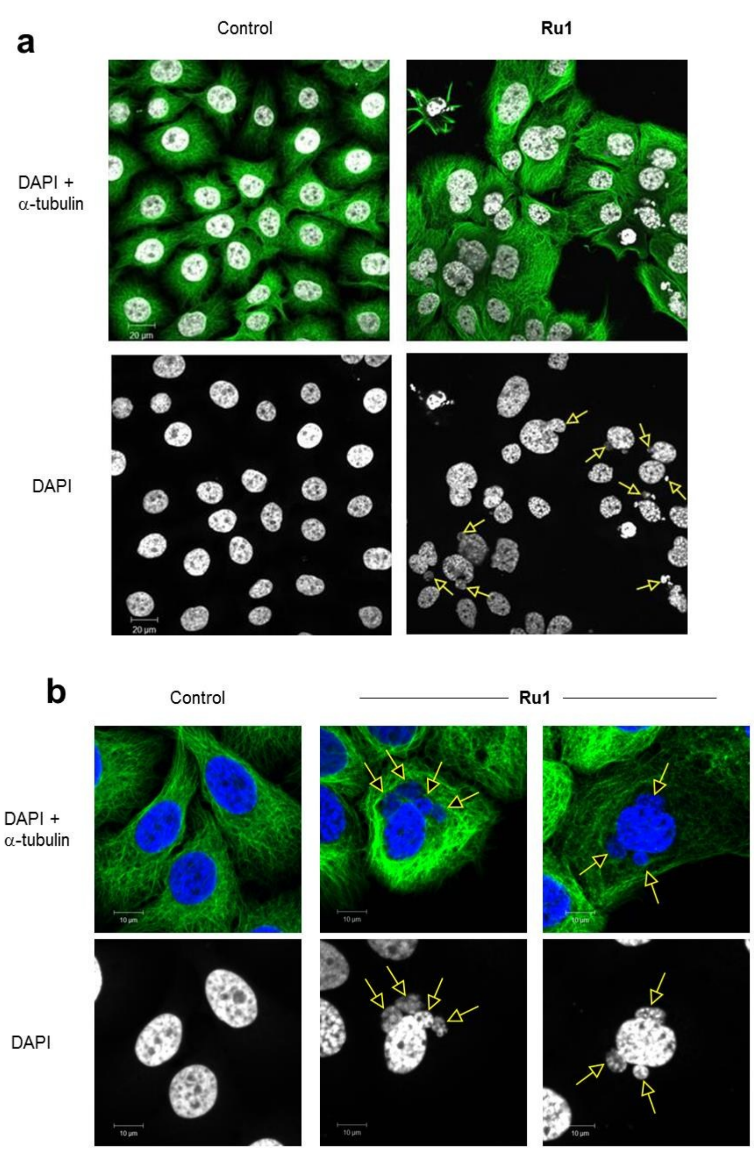

Figure S8. a) Identification of OE21 cells containing micronuclei (arrows) after treatment with **Ru1** (20 µM, 24 h). b) Higher magnification images showing OE21 cells possessing multiple micronuclei (arrows). Cells were immunostained for α-tubulin (green) and DNA (DAPI, blue or white).

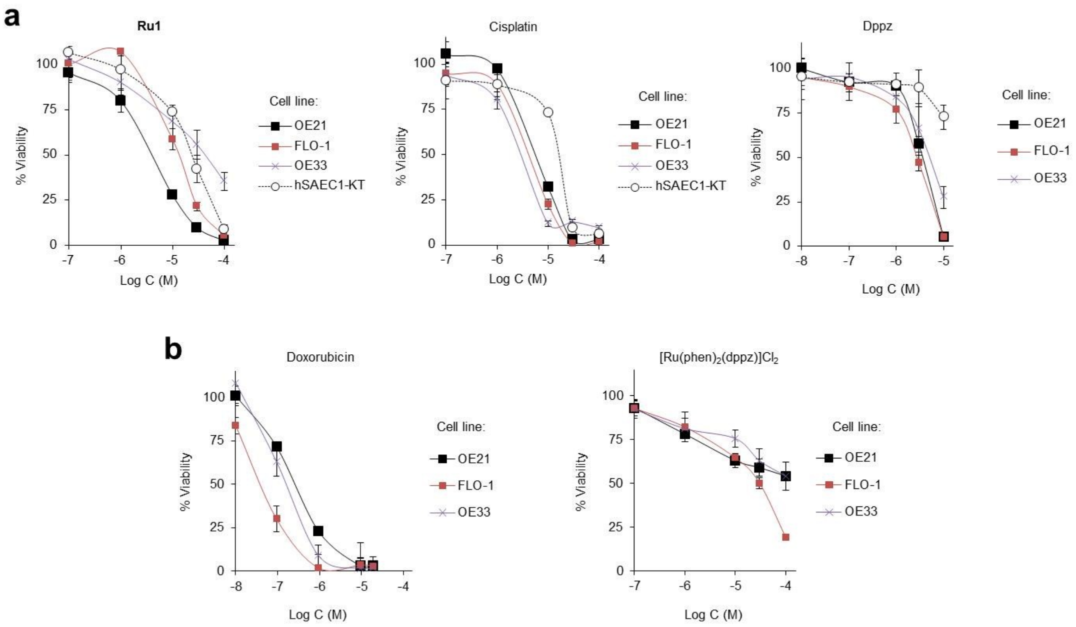

Figure S9. (a) Impact of **Ru1**, cisplatin or dppz on cell viability of OE21, FLO-1, OE33 oesophageal cancer cells or hSAEC1-KT immortalised normal human small airway epithelial cells, as determined by MTT assay (72 h constant incubation). (b) Impact of Doxorubicin or $[Ru(phen)_2(dppz)]Cl_2$ on cell viability of OE21, FLO-1, OE33 oesophageal cancer cells (72 h constant incubation). % viability determined by MTT assay and normalised to a negative control for each experiment. Mean of quadruplicates +/- S.D.. Representative of two independent experiments.

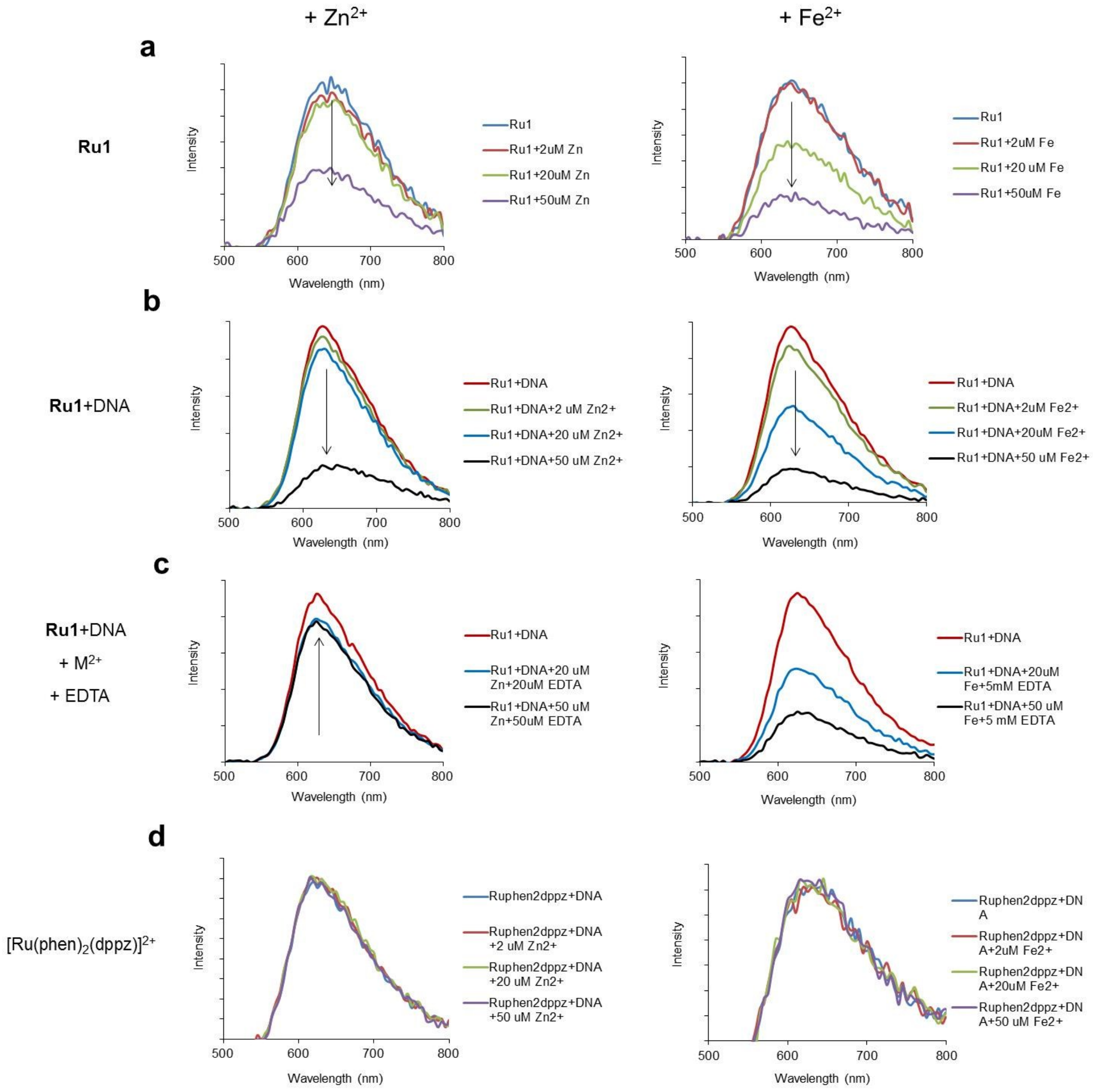

Figure S10. (a,b) Emission spectra (λ_{ex}=458 nm) of **Ru1** (2 μM) and **Ru1**+DNA solutions (2 μM **Ru1**, 0.2 ng/ml DNA) with increasing amounts of $ZnCl_2$ or $FeCl_2$ (2, 20 or 50 μM). Samples were mixed together at r.t. for 1 h. (c) Solutions from (b) with the subsequent addition of 20 μM EDTA (for Zn^{2+} treatment) or 5 mM EDTA (for Fe^{2+} treatment). (d) Emission spectra of [Ru(phen)2(dppz)]2+ + DNA solutions (10 μM [Ru(phen)2(dppz)]2+, 0.2 ng/ml DNA) with increasing amounts of $ZnCl_2$ or $FeCl_2$ (2, 20 or 50 μM). Experiments conducted in 5 mM Tris buffer pH 7.4 and all spectra were collected using the same instrumental conditions.

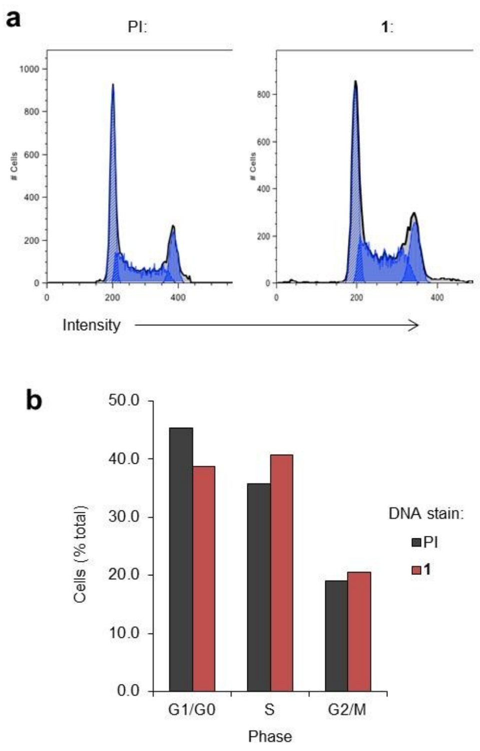

Figure S11. (a) Cell-cycle profiles of untreated OE21 cells stained for DNA content with either PI (5 μM, left) or **Ru1** (100 μM, right) after ethanol fixation, as determined by flow cytometry (FL2 channel). (b) Cell-cycle phase analysis showing comparable cell-cycle distribution for cells stained with either PI or **Ru1**.

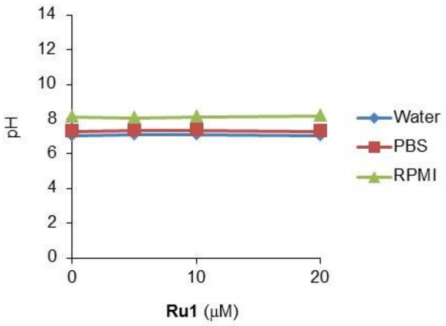

Figure S12. Impact of **Ru1** on pH of solutions employed in this study.

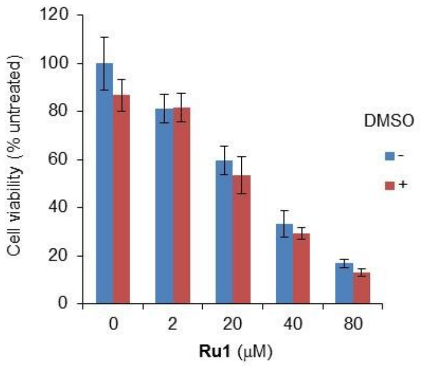

Figure S13. Impact of inclusion of DMSO (0.25%) in cell medium upon cell viability of FLO-1 cells treated with **Ru1** (32 h incubation). Average of quadruplicates +/- S.D. **Ru1** was used in DMSO-free media in all other experiments.

Supplementary References

1. J. Bolger, A. Gourdon, E. Ishow and J.-P. Launay, *Inorg. Chem.*, 1996, **35**, 2937.
2. A. E. Friedman, J. C. Chambron, J. P. Sauvage, N. J. Turro and J. K. Barton, *J. Am. Chem. Soc.*, 1990, **112**, 4960.
3. R. M. Hartshorn and J. K. Barton, *J. Am. Chem. Soc.*, 1992, **114**, 5919.
4. M. R. Gill, S. N. Harun, S. Halder, R. A. Boghozian, K. Ramadan, H. Ahmad and K. A. Vallis, *Sci. Rep.*, 2016, **6**, 31973.
5. B. Vaz, M. Popovic, J. A. Newman, J. Fielden, H. Aitkenhead, S. Halder, A. N. Singh, I. Vendrell, R. Fischer, I. Torrecilla, N. Drobnitzky, R. Freire, D. J. Amor, P. J. Lockhart, B. M. Kessler, G. W. McKenna, O. Gileadi and K. Ramadan, *Mol. Cell*, 2016, **64**, 704.
6. S. Timme, S. Ihde, C. D. Fichter, V. Waehle, L. Bogatyreva, K. Atanasov, I. Kohler, A. Schopflin, H. Geddert, G. Faller, D. Klimstra, L. Tang, T. Reinheckel, D. Hauschke, H. Busch, M. Boerries, M. Werner and S. Lassmann, *Oncogene*, 2014, **33**, 3256.
7. C. D. Fichter, C. Herz, C. Münch, O. G. Opitz, M. Werner and S. Lassmann, *BMC Cell Biology*, 2011, **12**, 13.
8. https://www.dsmz.de/catalogues/details/culture/ACC-698.html.
9. R. D. Ramirez, S. Sheridan, L. Girard, M. Sato, Y. Kim, J. Pollack, M. Peyton, Y. Zou, J. M. Kurie, J. M. DiMaio, S. Milchgrub, A. L. Smith, R. F. Souza, L. Gilbey, X. Zhang, K. Gandia, M. B. Vaughan, W. E. Wright, A. F. Gazdar, J. W. Shay and J. D. Minna, *Cancer Res.*, 2004, **64**, 9027.
